# Modeling sensitivity and uncertainties in platelet activation models applied on centrifugal pumps for extracorporeal life support

**DOI:** 10.1038/s41598-019-45121-2

**Published:** 2019-06-19

**Authors:** Gabriel Fuchs, Niclas Berg, L. Mikael Broman, Lisa Prahl Wittberg

**Affiliations:** 1 Department of Physiology and Pharmacology, Karolinska Institutet, Stockholm, Sweden; 2 Department of Cardiology, Sundsvall’s Hospital, Sundsvall, Sweden; 3 Linné Flow Centre & BioMEx, KTH Mechanics, Royal Institute of Technology (KTH), Stockholm, Sweden; 4 ECMO Centre Karolinska, Pediatric Perioperative Medicine and Intensive Care, Karolinska University Hospital, Stockholm, Sweden

**Keywords:** Cardiac device therapy, Translational research, Applied mathematics

## Abstract

Two platelet activation models were studied with respect to uncertainties of model parameters and variables. The sensitivity was assessed using two direct/deterministic approaches as well as the statistical Monte Carlo method. The first two, are linear in character whereas the latter is non-linear. The platelet activation models were applied on platelets moving within an extracorporeal centrifugal blood pump. The phenomenological, Lagrangian stress- and time-based power law-based models under consideration, have experimentally calibrated parameters and the stress expressed in a scalar form. The sensitivity of the model with respect to model parameters and the expression of the scalar stress was examined focusing on a smaller group of platelets associated with an elevated risk of activation. The results showed a high disparity between the models in terms of platelet activation state, found to depend on the platelets’ trajectory in the pump and the expression used for the scalar stress. Monte Carlo statistics was applied to the platelets at risk for activation and not to the entire platelet population. The method reveals the non-linear sensitivity of the activation models. The results imply that power-law based models have a restricted range of validity. The conclusions of this study apply to both platelet activation and hemolysis models.

## Introduction

Development of artificial support of organ function or replacement of organs has been an important objective for a long time. The main advantage with artificial organs is the almost unlimited availability. Thus, the shortage of human organs and risk for the recipient concerning organ rejection can be circumvented. On the other hand, although sophisticated, artificial organs are still considerably less efficient than their natural counterparts. More importantly, artificial organs for circulatory support are associated with thromboembolic, pro-inflammatory and hemolytic complications. Also in cardio-respiratory support that is limited in time (from less than a week to months), i.e. extracorporeal membrane oxygenation (ECMO), common problems are thrombus formation^1^ and increased risk of patient bleeding^2^. Such complications are the main reason why organ transplant or organic components are most often preferred. Platelet adhesion plays a central role in hemostasis and activation occurs at sites of vascular wall injury. The initiation starts by adhesion to macromolecules, such as collagen and von Willebrand factor (vWF), or platelet agonists, such as ADP, thrombin, and thromboxane A2^3^. Furthermore, artificial surfaces may act as initiators for platelet activation high- and low-shear^4^. When coated by a heparin-based lining reducing thrombin and factor X activation^5^, platelet activation induced by the artificial surfaces lessens. However, this measure does not completely eliminate the rate of complications^4^. The failure is due to multiple activation pathways and thereby ensuring the functionality of the coagulation system. Premature platelet activation may lead to heparin induced thrombocytopenia^6^.

Platelet activation is most often induced through heparin independent mechanisms. One such mechanism is the mechanical forces acting on the platelets. Recent research indicates that platelets are able to respond to mechanical stress through mechano-sensing. Qiu *et al*.^7^ argue that platelets sense the stiffness of the underlying substrate (fibrin/fibrinogen) inducing platelet adhesion and spreading. Zhang *et al*.^8^ show mechano-sensing to be anisotropic, i.e. directional dependent. Furthermore, experiments suggest that platelet integrins require lateral forces to mediate platelet–platelet interactions. Chopard *et al*.^9^ studied *in-vitro* and developed a numerical model to improve the understanding of the role of directional sensing in platelet adhesion and aggregation. Additional work on mechano-sensing reported the platelet aggregation process to occur in multiple steps, out of which all except two specific steps were reversible^10^.

To assess platelet activation, mathematical models have been developed. Current platelet activation models do not account for the findings mentioned above. Instead, the models consider the magnitude of the stress, the accumulated stress effect, and some also the time-rate of change in stress. In a recent paper^11^, we employed the model of Nobili *et al*.^12^ to different components of the ECMO circuit. Numerical simulations of the flow in centrifugal blood pumps^13^ indicate that the platelets experienced varying stress in space and time that may significantly differ from the stress character in the calibration experiments^14,15^. Further, Hemodynamic Shearing Device (HSD)^16,17^ experiments used to calibrate the platelet activation models of Soares’ and Consolo explicitly includes the temporal rate of change of the stress^12,14^. However, for flows deviating from the calibrated conditions, validation of platelet activation models remains difficult. This is mainly due to the lack of detailed flow information in terms of the temporal and spatial variations of the stress tensor components and blood constituent distribution.

Regarding mechanical stress acting on blood cells, it has been assumed to lead to cell deformation and ultimately to cell damage. Moreover, it has been rather common to assert that cell damage depends on the strength of the stress and its duration^18^. This idea has been expressed in terms of power law formulation, relating the damage (*D*) to the stress (σ) and exposure time (*t*) through a simple relation: *D* = *C* σ^α^ *t*^β^. This concept has been applied to damage of red blood cells (RBC)^19^, white blood cells^20^ and platelets^21^. The platelet activation models of Nobili and Soares are congruent with the RBC damage models. These models include the stress and time as well as three or six model parameters in the Nobili and Soares activation models, respectively. The multiple path and complex regulatory processes controlling the coagulation system implies that for the foreseeable future, it will be impossible to account for all the different chemical and physical details of the involved processes. Thus, the goal of the models has been to provide a (semi-) quantitative understanding of where and when activation of platelets may occur. The models under consideration were tested and calibrated under time-dependent but relatively low-stress conditions. The flow in the centrifugal pump that was used in this study contains temporally varying low- and high-stress regions. The mechanism of clot formation at high-shear differs from low-shear conditions^22^. Hastings *et al*.^23^ considered the location and composition of thrombosis at high-shear. It is rather clear that there is need to assess the validity and sensitivity of promising platelet activation models to operating conditions that may differ significantly for the calibrating conditions. Determining the sensitivity of a model to errors/uncertainties in model parameters is a way for assessing model applicability for a particular application. The flow in the ECMO pump is challenging and offers a range of stress compositions (i.e. anisotropy) that the platelet encounters when in the pump^11,13^.

The purpose of this work was to increase the understanding of the strengths and shortcomings of the platelet activation models under consideration by quantifying the sources for uncertainties, i.e. the errors of the parameters and model definition variables. Sensitivity and uncertainty analyses were carried out by considering derivatives of the respective parameters, assessment of the individual contribution from the different terms appearing in the models as well as model response to perturbations. Two different flow setups were studied; a uniform stress field investigating the sensitivity of the different platelet activation models under steady as well as time-depended stress and the activation model sensitivity for the flow in a centrifugal blood pump.

## Numerical Methods and Platelet Activation Models

The models under consideration are described in the following along with the methods used in order to analyze the associated model uncertainties. First, the numerical approach applied to solve for the flow in an ECMO centrifugal blood pump is shortly presented. Thereafter, the methods for analyzing the platelet activation models’ sensitivity are discussed.

### Flow simulation - centrifugal blood pump

The flow was simulated by solving the conservation laws for mass and momentum, assuming that the fluid is Newtonian, and the flow incompressible and isothermal. Under these assumptions, the governing equations are as follows:

1 $$\frac{\partial {u}_{i}}{\partial {x}_{i}}=0$$

2 $$\frac{\partial {u}_{i}}{\partial t}+\frac{\partial ({u}_{i}{u}_{j})}{\partial {x}_{j}}=-\frac{1}{\rho }\frac{\partial p}{\partial {x}_{i}}+\frac{\partial }{\partial {x}_{j}}\nu \frac{\partial \rho {u}_{i}}{\partial {x}_{j}}$$

where ρ denotes the density, ν is the kinematic viscosity, *p* the pressure and *u*_*i*_ the fluid velocity component in the *i-th* direction. The governing equations are valid for laminar, transitional and turbulent flows, provided that the flow details are resolved, i.e. all length and time-scales encountered in the flow. In this work, no explicit turbulence model was applied. The fluid had a constant kinematic viscosity ν = 0.9 × 10^−6^ m^2^/s and density of 998 kg/m^3^. A sliding mesh technique was employed to account for the rotation of the impeller of the centrifugal pump. The governing equations were discretized on a polyhedral grid by a formal second order finite-volume method. Both inlet and outlet were extended by adding a straight pipe having a length of 20 and 15 diameters, respectively. At the inlet boundary, the top-hat velocity profile (constant axial component and vanishing in-plane velocity components) yields a specified flow rate. A constant pressure was assumed, implying a parallel flow at the outlet. For the solid walls, no-slip conditions were applied. Being the most appropriate in order to solve for flows including regions of laminar, turbulent and transitional flows, Large Eddy Simulation (LES) was employed. However, this requires the spatial resolution to be high enough to resolve turbulent regions, at least a portion of the inertial sub-range. Under these conditions, there is no need to explicitly add a sub-grid scale model. Further details of the numerical method and the features of the flow field in the centrifugal pump is found in Berg *et al*.^13^.

The flow field was computed during a period long enough to minimize the effects of initial conditions, achieved after approximately 30 pump revolutions. Thereafter, the platelet motion and the flow data along the platelet path were sampled for later analysis. A large number of platelets (10^5^ in most cases) were released at the inlet section and randomly distributed at the initial position plane. The motion of the platelets was computed by integrating equation (3) using the trapezoidal rule. The platelets were tracked while moving in the fluid, being exposed to shear and normal-stresses by the fluid. The large-scale effect of these stresses constitutes the drag forces, whereas the small-scale effect on the platelet is possible platelet distortion and ultimately platelet activation.

Using platelet activation approaches as applied here includes further assumptions on the fluid as well as the flow. As the platelet volume fraction (i.e. platelet volume in a given region relative to the volume of the region) is small, the platelets are uniformly distributed. The mean distance between the platelets is large as compared to platelet size (by a factor of about 10). Hence, platelet-to-platelet interaction may be neglected enabling individual tracking of platelets and, when relevant, platelet-to-platelet interaction is indirect (i.e. through interaction with the carrier fluid). As the size of the platelets is small (order of 2 μm) the platelets follow the carrier fluid. Furthermore, the platelets are assumed to behave as rigid spheres. The relevant parameters can be expressed quantitatively in terms of dimensionless Stokes (*S*_*t*_) number based on the platelet relaxation time (O(10^−7^)) and flow time (O(10^−3^) (*S*_*t*_ = O (10^−2^)-O (10^−4^)). In order to follow the platelets, Lagrangian Particle Tracking (LPT) was used, integrating the platelet motion:

3 $${m}_{p}\frac{d{u}_{p}}{dt}={F}_{p;}\frac{d{x}_{p}}{dt}={u}_{p}$$

where *m*_*p*_, *u*_*p*_, *x*_*p*_ and *F*_*p*_ are the mass, velocity vector, location and the force acting on the platelet, respectively. Due to the small platelet Reynolds number (O(10^−3^), assuming a slip velocity of less than 0.1% of the bulk flow), it is possible to assume that the only force acting on the platelets is Stokes drag. Furthermore, as the platelet volume fraction is low, the force the platelet exerts on the fluid (equal to the force that the fluid exerts on platelets) is small and can thus, be neglected. Altogether, these assumptions result in a one-way interaction between fluid and platelet.

### Activation models and Parameter sensitivity assessment

The platelet activation models considered in this analysis, namely that of Nobili, Soares and Consolo, are explained in detail in multiple papers^12,14,15,21^. In the following, a shorter description is provided.

Nobili’s model for platelet activation uses PAS as dependent variable. Platelet activation state is a parameter that varies between 0 and 1 for a population of platelets. “Zero” implies no activated platelets and “One” implies full activation of the platelet population. The model assumes PAS to be dependent upon two variables: the scalar stress (τ) and residence time (t). Platelets immersed in the fluid are subject to a stress that is a force in a direction *i* acting on a surface with a normal in the *j*-direction. The stress is a symmetric tensor with components τ_ij_. The Nobili’s platelet activation model uses a scalar form of the stress, in the following denoted by τ, without indices;

4 $$\frac{d\,PAS}{dt}=ca{\tau }^{b/a}{D}^{a-1}\,;\,\frac{d\,D}{dt}={\tau }^{b/a}$$

This formulation has three model parameters: *a, b*, and *c* where the numerical values were determined by measurements using the Hemodynamic Shearing Device (HSD)^16^. In the experiments, the only non-vanishing component of the stress tensor had one shear component. In general, the scalar stress can be defined by different formulations. For modeling of hemolysis, Yu *et al*.^24^ considered several formulations for the scalar stress. A simple formulation of the scalar stress, is the scalar product of the stress tensor, i.e. τ^2^ = τ_ij_ τ_ji_/2. Other formulations have been proposed and some are used herein (cf. equation (12)). Consequently, different formulations of the scalar stress of the given stress tensor often results in different PAS values.

Soares *et al*.^14^ proposed a model that, in addition to the above parameters, also includes the effects of non-steady stress that may act on the platelets. Although having a starting point similar to other power law formulations, it allows for effects contributable to platelet activation in an additive manner.

5 $$\frac{d\,PAS}{dt}={K}_{0}(1-PAS)$$

where *K*_0_ is the function of PAS itself and the stress τ is calculated along the platelet path. In other words, *K*_0_ is a function of time and the path along which the platelet moves. For a constant *K*_0_, PAS vanishes at time zero. However, PAS increases exponentially and approaches asymptotically a final value of PAS = 1 with time. Here, *K*_0_ is assumed to be composed of three contributions: *S*, *F* and *G*, all functions of time and the stress along the platelet path. *S* and *F* are closely related to the Nobili model. The S-term is a contribution due to the history of the stresses that acted on the platelet. The variable *D* in the Nobili’s model, with *b/a* = 1, renamed as *H*, implies that

6 $$H(t)={\int }_{0}^{t}\tau (s)\,ds$$

Thus, H is an expression of the stress accumulation (SA) along the path of the platelet. Soares *et al*.^14^ assert that the stress history contribution, denoted by S, is given as (with *S*_*r*_ being a model parameter):

7 $$S(PAS,H)={S}_{r}PAS(t)H(t)$$

The power law model also explicitly contains the instantaneous local stress contribution, which in the model of Soares^14^ is expressed as

8 $$F(PAS,\tau )={C}^{1/\beta }\beta \,PAS{(t)}^{\frac{\beta -1}{\beta }}\tau {(t)}^{\frac{\alpha }{\beta }}$$

The third term G added to the model expresses the temporal variations of the affected platelet’s response. Thus, *G* is a function of the averaged time derivative of the stress (i.e. Stress Rate, SR).

9 $$G(PAS,\mathop{\tau }\limits^{\bullet })={C}_{r}^{1/\delta }\delta \,PAS{(t)}^{\frac{\delta -1}{\delta }}\mathop{\tau }\limits^{\bullet }{(t)}^{\frac{\gamma }{\delta }}$$

The final form of this model is given by

10 $$\frac{d\,PAS}{dt}=(S+F+G)\,(1-PAS)$$

The model as described by Soares *et al*.^14^ and Consolo *et al*.^15^, defines the stress-rate as the *average* of the absolute value of time-derivative of the stress (SR in equation (13)). Note that all variables including the time derivative are computed along the platelet path (Lagrangian frame) in contrast to the variables and derivatives computed in the governing Eqs. 1 and 2, computed in the Eulerian frame.

Both models (equations (4 and 10)) were calibrated by determining the numerical values of the model parameters by fitting to measured data^12,14,15^, where the model parameters for equations (4–9) are provided in Table 1. Consolo’s model parameters differ from those of Soares in four of the seven parameters.

**Table 1:** Model parameters (Nobili: equation (5); Soares equations (7–9), and Consolo: equations (7–9).

	*a*	*b*	*c*	S_*r*_	C	α	β	C_*r*_	γ	δ
Nobili^12^	1.3198	0.6256	10^−5^							
Soares^14^				1.5701 10^−7^	1.4854 × 10^−7^	1.4854	1.4401	1.3889 10^−4^	0.5720	0.5125
Consolo^15^				1.5701 10^−7^	1.4854 × 10^−7^	0.71825	0.72005	2.78 10^−5^	0.75	0.5125

It may be noted that due to the non-linearity of equation (10), the qualitative assessment of the behavior of equation (5) does not apply. The optimized parameters proposed by Consolo *et al*.^15^ implied a substantial decrease in *C*_*r*_ and in β (by a factor of 5 and 2, respectively) and an increase in γ by about 30%. The impact of the optimized parameters is that the F-term increases its contribution to PAS (stronger non-linearity) whereas the weight to the scalar stress is practically maintained. On the other hand, the modified γ value implies an increased weight to the time-rate of change of the stress. Since all model parameters values are provided with four to five digits of accuracy, analysis of sensitivity of the models regarding the different variables is reasonable.

### Model sensitivity analysis

In order to analyze the associated model uncertainties, four different approaches were applied;

- Sensitivity (derivatives) of PAS with respect to the parameters.
- Assessment of the contribution of different terms in each model and relating these to the stress encountered by individual platelets.
- Monte-Carlo (MC) perturbations of model parameters. The outcome was assessed with respect to PAS variations due to collective and individual random perturbation of the model parameters.
- The sensitivity of the models was assessed with respect to platelet motion, encountered stress, stress accumulation (SA), averaged stress rate (SR), stress spectra and platelet residence times.

The post-processing in the current work was performed using MATLAB scripts to compute PAS by the activation models and to compute the sensitivity of the model parameters.

### Assessment of sensitivity to changes in model parameters

To estimate the sensitivity of the results to changes in the parameters, the first derivatives of the results with respect to the parameters under consideration were computed. With the assumption that the dependence of the results on the parameters is smooth, the first derivatives (i.e. the leading term in the Taylor expansion) provide an estimate of the sensitivity of the results to uncertainties and errors. For example, taking the derivatives of equation (4) with respect to the model parameters *a, b* and *c*, yields the following:

11a $$\frac{d}{dt}(\frac{\partial \,PAS}{\partial a})=c\,{\tau }^{b/a}\,{D}^{a-1}[-\frac{b\,\mathrm{ln}\,\tau }{{a}^{2}}+1+a\,\mathrm{ln}\,D+a\frac{a-1}{D}\frac{\partial D}{\partial a}]$$

11b $$\frac{d}{dt}(\frac{\partial \,PAS}{\partial b})=c\,{\tau }^{b/a}\,{D}^{a-1}[\mathrm{ln}\,\tau +a\frac{a-1}{D}\frac{\partial D}{\partial b}]$$

11c $$\frac{d}{dt}(\frac{\partial \,PAS}{\partial c})=a\,{\tau }^{b/a}{D}^{a-1}$$

11d $$\frac{d}{dt}(\frac{\partial \,D}{\partial a})=-\,\frac{b{\tau }^{b/a}\,\mathrm{ln}\,\tau }{{a}^{2}}$$

11e $$\frac{d}{dt}(\frac{\partial \,D}{\partial b})=\frac{{\tau }^{b/a}\,\mathrm{ln}\,\tau }{a}$$

In addition to the sensitivity to model parameters, the corresponding sensitivity to the scalar stress is considered.

11f $$\frac{d}{dt}(\frac{\partial \,PAS}{\partial \tau })=c\,a{\tau }^{b/a}{D}^{a-1}[\frac{1}{\tau }+\frac{1}{D}\frac{\partial D}{\partial \tau }]\,;\,\frac{d}{dt}(\frac{\partial \,D}{\partial \tau })={\tau }^{(b-a)/a}\,$$

Equation (11) were integrated along the platelet pathline, as carried out for equation (4). Moreover, equation (11f) can be useful in order to assess the effects of turbulence and the modeling thereof, as commonly done in Reynolds averaged formulations for turbulence (RANS).

### Assessment of terms in the models

Computing the magnitude of the different terms may provide an *a posteriori* estimate of the different (driving) terms of the models. For example, the information acquired by calculating the *D*^*(a*−*1)*^ term in the Nobili’s model in space and time could be correlated to the flow and stress the platelet experiences. Similarly, the three terms on the right side of equation (10) (S-, F- and the G-terms) were computed and their response to the local stress could be assessed. The importance of each term accentuates the issues related to the model and/or the reasons for the fluid mechanical risks for platelet activation. The relative importance of the different terms needs to reflect the underlying concepts and understanding of platelet activation; in turn an approach helpful in detecting apparent model inconsistencies.

### Assessment of Monte-Carlo statistics

The global sensitivity of a model can be assessed by introducing an uncertainty in the model parameters and/or variables. Assuming a normal distribution of a specified amplitude, perturbations on the model parameters were applied, after which PAS was computed. When the number of computed cases is large enough, it becomes meaningful to compute the mean and variance of the PAS results, enabling determination of the sensitivity as well as distribution of the outcome of the model. This approach is simpler to implement as compared the alternatives described above. However, it requires significantly larger computational effort, but with the main advantage of being able to implicitly handle model non-linearities. By taking large values of uncertainties/errors, an improved assessment of potential effects of model nonlinearity can be attained. During the integration of the platelet along its path, the model parameters were perturbed by a factor derived from a normal distribution with a pre-determined amplitude (10%, 20% & and 30%, respectively). This process was repeated for each platelet a large number of times to reach the sample size for a given statistical error of the mean. The required sample size can be assessed as part of the simulation. For a required standard error of the mean of ε, *N* samples must be calculated; such that N = O((σ/ε)^2^), where σ is the standard deviation. In our computations we used 250 samples.

### Scalar stress effects

The effect of the stresses on the platelets are most probably dependent on the type of the stress^7^. Normal- and shear-stress components could lead to different response. However, as no such experimental data is available, several scalar expressions (instead of the six components of the symmetric stress tensor) were considered. The models under consideration depend on the expression for the (scalar) stress. The stress tensor can be contracted in different ways, depending on the application. Three different expressions for the stress were considered to demonstrate the impact of the scale stress definition on the outcome of the activation models.

12 $${\tau }_{a}=\sqrt{\frac{1}{6}[{({\tau }_{ii}-{\tau }_{jj})}^{2}+{\tau }_{ij}^{2}]}\,;\,{\tau }_{b}=\sqrt{\frac{1}{3}{\tau }_{ij}\,{\tau }_{ji}}\,i\ne j\,;\,{\tau }_{c}=\sqrt{\frac{1}{3}{\tau }_{ii}^{2}}$$

The expression for *τ*_*a*_ has commonly been used for expressing the scalar stress^11^. The other two expressions involve the shear-stress components (*τ*_*b*_) or the normal-stress components (*τ*_*c*_), respectively. These three stresses were used to demonstrate the impact of the scale stress definition on the outcome of the activation models. The scalar stresses were computed along the paths of the platelets. Thus, when considering the time variation of the scalar stress, the time-derivative is taken in this (Lagrangian) framework, which differs from the common time-derivative of the stress in a stationary frame (Eulerian). Here, we refer only to the former time-derivative of the stress.

Two measures to characterize the scalar stress were proposed^14^: Stress Accumulation (SA) effect (expressed as the discrete form of *H* in equation (6)), and Stress Rate (SR). These parameters are defined in terms of the time-dependent scalar stress (*τ*(*t*_*i*_)) at a set of discrete times *t*_*i*_.

13 $$SA=\sum _{i=1}^{N}\frac{\tau ({t}_{i+1})+\tau ({t}_{i})}{2}{\rm{\Delta }}t\,;\,SR=\frac{1}{N}\sum _{i=1}^{N}\frac{|\tau ({t}_{i+1})-\tau ({t}_{i})|}{\Delta t}$$

where *N* is the number of time-steps. *Δt* is the time interval between two consecutive time steps (i.e. *Δt* = *t*_*i+1*_ *−* *t*_*i*_). Thus, SR amounts to a second order approximation to the mean absolute value of the time derivative of the scalar stress. As indicated in^14,15^, the Soares’ model uses SR and not the instantaneous time derivative of the local stress.

## Results

Firstly, cases with platelets subjected to a uniform stress throughout the simulation were investigated. The sensitivity of the different platelets to activation models under steady and time-dependent stress were assessed. Thereafter, the behavior of the activation models was studied for the flow in a centrifugal blood pump.

### Uniform stress field

**Figure 1:**
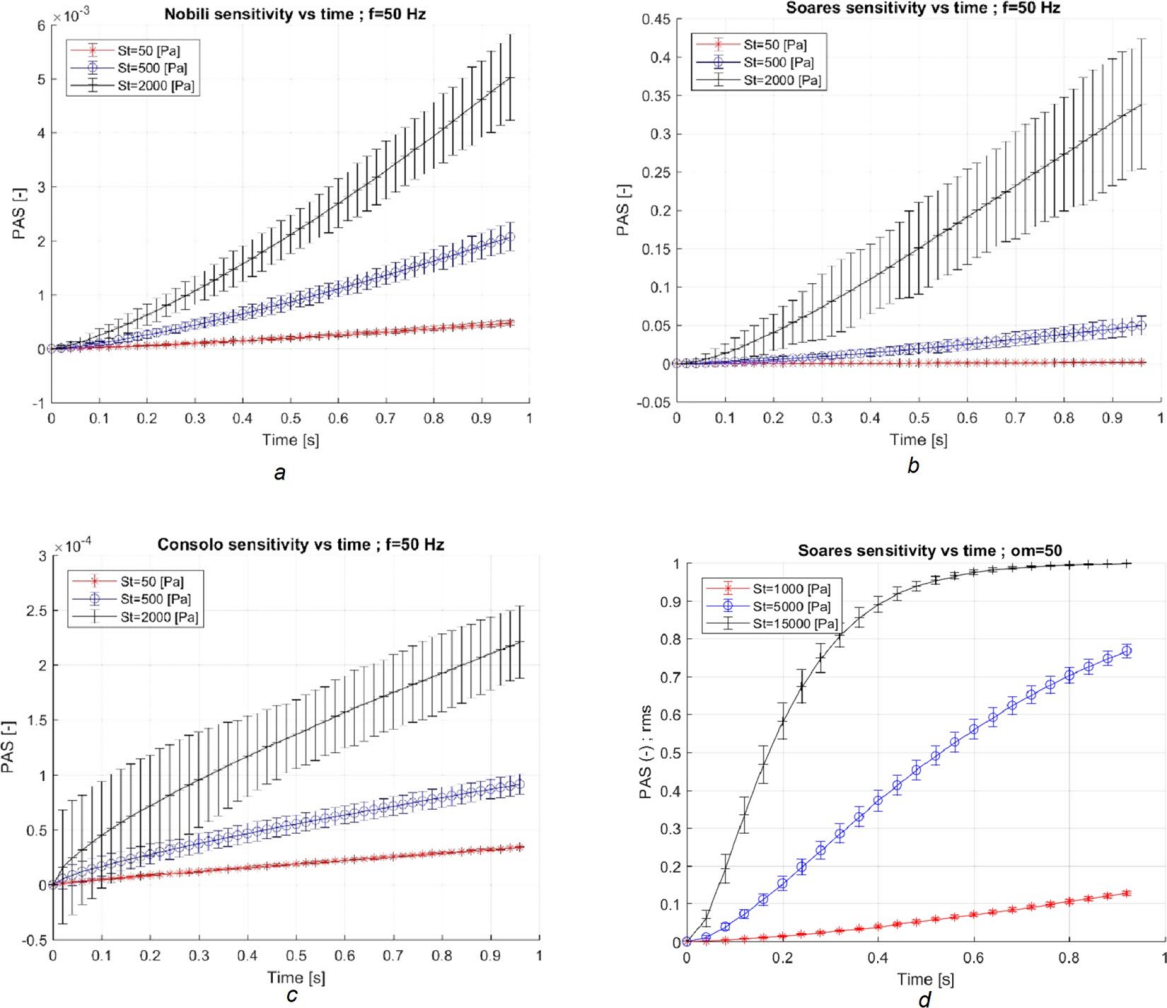
PAS vs. time using Nobili’s (**a**), Soares’ (**b**) and Consolo’s (**c**) models, respectively. A uniform stress field in the range of 5–2,000 Pa with periodic oscillations at 50 Hz and amplitude of 10% was used. The integration time was limited to 1 s. The activation models exhibit a monotone increase in PAS with time and the strength of the stress. The Soares’ model predicts largest PAS value at the end of the simulation time (1 s), followed by Nobili’s model that is smaller by more than one order of magnitude. Moreover, Consolo’s model predicts a PAS that is smaller by more than one order of magnitude as compared to Nobili’s model. Also, the initial slopes (time-derivative of PAS with time) is increasing at least during the first second (a and b). Consolo’s model has a different initial behavior due to the value of a model parameter. To assess the asymptotic behavior of the models, an elevated stress (15 kPa) was applied for 1 s (**d**). After 1 s the Soares’ model predicts a complete activation of the platelet population. The PAS response of the models to random perturbations of 10% amplitude is marked by error bars. The uncertainty in PAS increases with PAS itself, except when PAS reaches large values, asymptotically approaching 1. Of note is also the large, almost uniform sized, error bars computed by Consolo’s model for τ = 2 kPa.

Figure 1 depicts the development of Platelet Activation State (PAS) in time for the different, time-independent and dependent stresses for different model set-ups in the range of 5 Pa to 15 kPa. Both Nobili’s and the original Soares’ models exhibited a non-linear growth over time and the PAS level increase with increasing stress. There were clear and significant differences between the models, as detailed in Fig. 1. For example, the Soares’ model predicted about 35% of the platelets to be activated, after 1 s, when subjected to a stress of 2 kPa. The corresponding number of platelets was about 0.5% (Fig. 1a) and 0.02% (Fig. 1c) with Nobili’s and Consolo’s models, respectively. The differences between the models are more pronounced with increasing stress (15 kPa). The original Soares’ model predicted a 100% platelet activation after about 1 s (Fig. 1d). The corresponding PAS value with Nobili’s model was only 2% (not shown). Moreover, Fig. 1 shows PAS response to random perturbations of the parameters of the models and stress field. Two hundred fifty random perturbations were introduced to the different model parameters. With increased level of stress, the models approached the asymptotic PAS value of 1, and consequently the level of uncertainty diminished.

**Figure 2:**
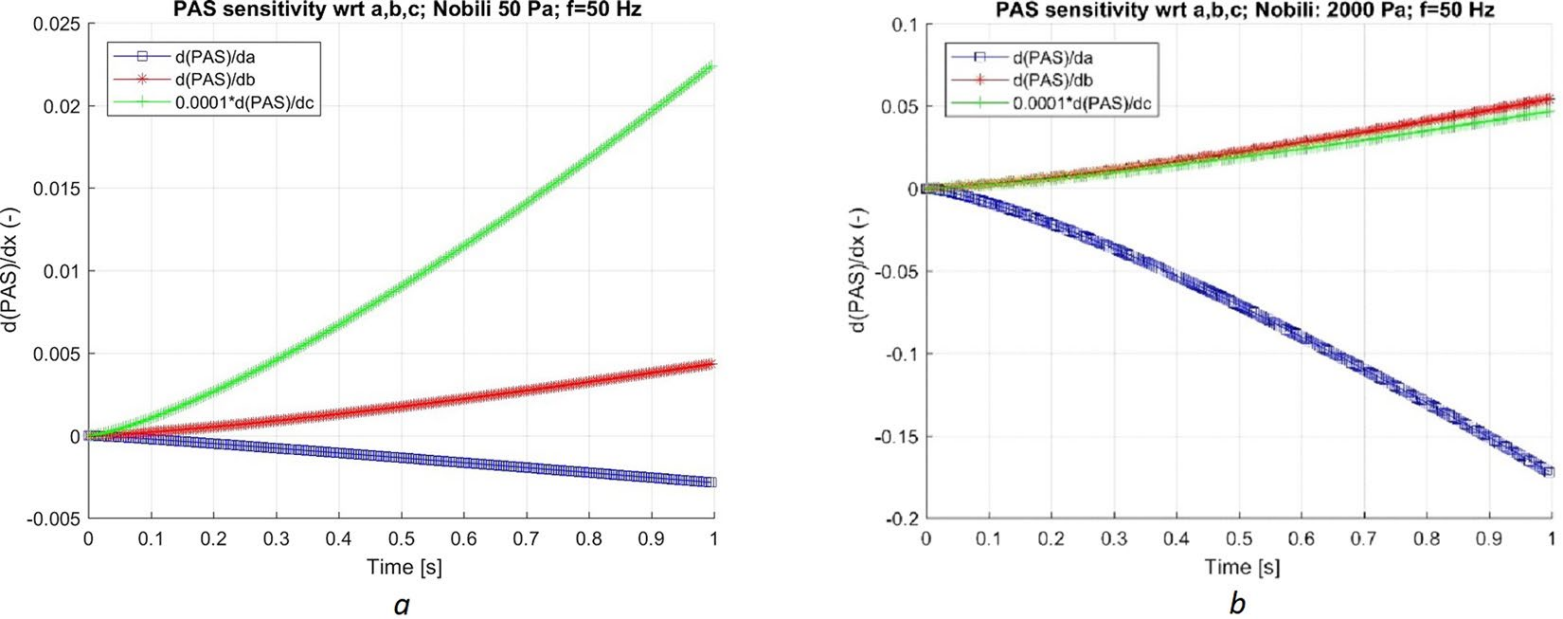
The sensitivity of Nobili’s model ($\partial PAS/\partial \zeta$) with respect to the three model parameters, with ζ = *a*, *b* or *c* (in equation (4)). The oscillating stress (t 50 Hz) was 50 Pa (**a**) and 2 kPa (**b**), respectively. Note the difference in the scales of the y-axis. Note also the downscaling of the PAS derivative with respect to *c* by a factor of 0.0001. The derivative of PAS with respect to *a* is negative while it is positive with respect to the other two parameters, *b* and *c*, indicating that cancellation effects may occur.

Studying the linearized sensitivity analysis of this simple case, Fig. 2 depict the sensitivity for constant stress of 50 Pa and 2 kPa, showing the derivative of PAS with respect to the model parameters. These derivatives are computed by integrating equation (11a–e) in time. Interestingly, PAS reduces with *a*, but increases monotonically with *b* and *c*. Thus, a cancelation effect of errors could occur. The trend of the sensitivity of the stresses was similar for the different stress levels. However, the magnitude of the sensitivity increased faster for parameter *a* as compared to *b* and *c*. At the end of the simulation (1 s), the value of PAS (Fig. 1a) for the 50 Pa case and the 2 kPa case were approximately 5∙10^−4^ and 5∙ 10^−3^, respectively. For the 50 Pa case, an error of 100% in the final PAS values would be attained (with only the first derivative was accounted for) with an error of about 20%, 7% and 20% in *a, b* and *c*, respectively. For the higher stresses the corresponding levels of uncertainty are about 3%, 15% and 10% perturbations in *a, b* and *c*, respectively. In absolute value terms, the most sensitive parameter is *c*, a scaling factor appearing in equation (4). With increasing stress, parameters *a* and *b* exchange places with respect to sensitivity and uncertainty. The *b* parameter is more important at lower stress whereas parameter *a* is more influential at higher stress. As *a* and *b* act in opposite directions, increasing both parameters may lead to an increase or decrease in PAS. Altogether, the linear response demonstrates the sensitivity of the platelet models to uncertainty in the model parameters with increasing integration in time.

The relative contributions of the D-term in Nobili’s model and the S and F-terms in the Soares’ and Consolo’s models were evaluated (not shown). The relative size of the D-term is smaller as compared to the H-term due to the exponent of τ (*b/a* = 0.47 and 1, for the Nobile and Soares models, respectively). Both the S and the F-terms increased in magnitude in Soares’ and Consolo’s models. For the former model, the relative size of the F-term was found to be almost two orders of magnitudes greater than the corresponding S-term. For Consolo’s model, the F-term was greater as compared to the corresponding S-term, but only by about one order of magnitude. These differences can be attributed to the parameter differences between the two models. Thus, these changes indicate the sensitivity of PAS to the numerical values of the model parameters.

### Platelet activation in a centrifugal blood pump

In contrast to the simple cases considered above, blood flow in natural or artificial organs/components is subject to stress characteristics that greatly change in space and time. The flow in a centrifugal blood pump of the type used in ECMO circuits has regions of widely varying stress, in terms of size as well as temporal variation of the stress-tensor components.

**Figure 3:**
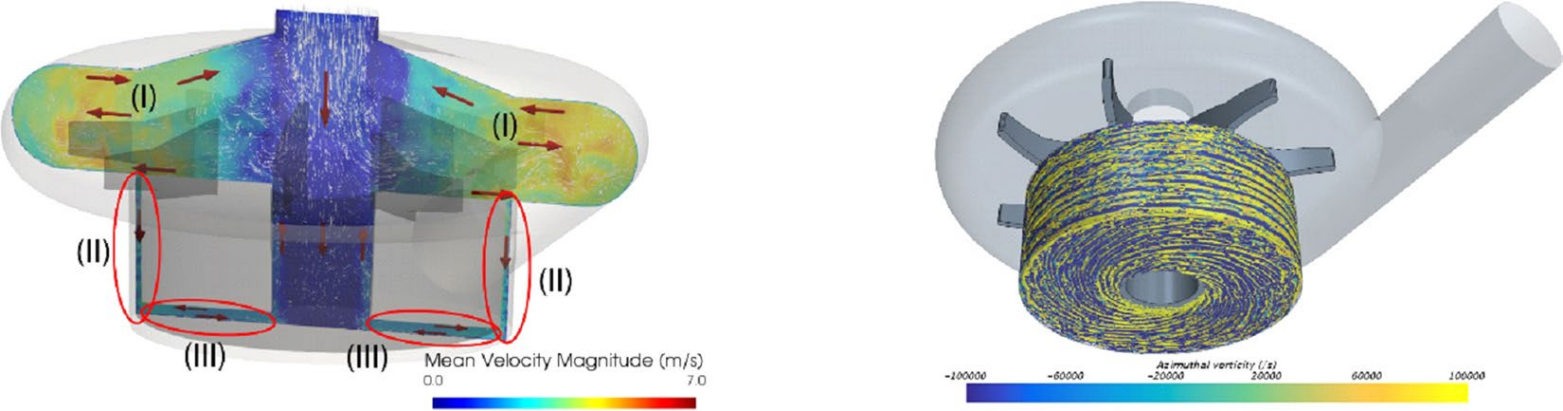
The mean flow field in the centrifugal pump (left) and the azimuthal vorticity within the gaps between the pump-house and the outer walls of the pump (right). In the left frame, Regions I–III depict the areas relevant to long platelet residence times. These include the re-circulating flow above the impeller (I), the gap between the rotating magnet house and the side walls (pump house), marked as Region II (Taylor-Couette like flow) and the bottom gap (III) with structures similar to those found in the Ekman and Bödewadt flows^13^). In the vortices in Regions II and III, platelets are captured and residence time prolonged^13^). Residence time added to the high shear generated in the narrow gap between the rotating magnet house and the pump house, induces a high risk for platelet activation.

The pump impeller considered here has a diameter of approximately 44 mm, consisting of four larger and four smaller blades. A magnetic field drives the impeller and maintains the combined unit of the magnet house and impeller levitating in the blood stream inside the pump housing at a desired position while rotating. The annular magnet house has a central hole of 9 mm in diameter, and a height of 12 mm. The impeller is placed in the pump house leaving a gap of 0.5 mm in radial and 1 mm axial direction between the impeller magnet and the pump house. The data presented here is obtained at a pump speed of 3,000 rpm (50 *Hz*) pumping 4 L/min. Figure 3 depicts the mean flow fields of three prioritized regions for platelet activation and model sensitivity, marked Regions I to III.

The flow field in the pump is unsteady, including both coherent, larger scale structures along with turbulent fluctuations^13^. The mean flow is characterized by several distinct recirculation regions. Figure 3b depicts the azimuthal vorticity component in Regions II and III. These regions are characterized by relatively slowly moving sets of vortices, in which platelets are captured, and remain trapped over a longer time. Details of the impact of these vortices on the activation of a platelet population were discussed by Fuchs *et al*.^11^. In the same paper, it was observed that the sensitivity of the results to the model parameters proposed by Nobili required additional analysis with respect to model sensitivity.

**Figure 4:**
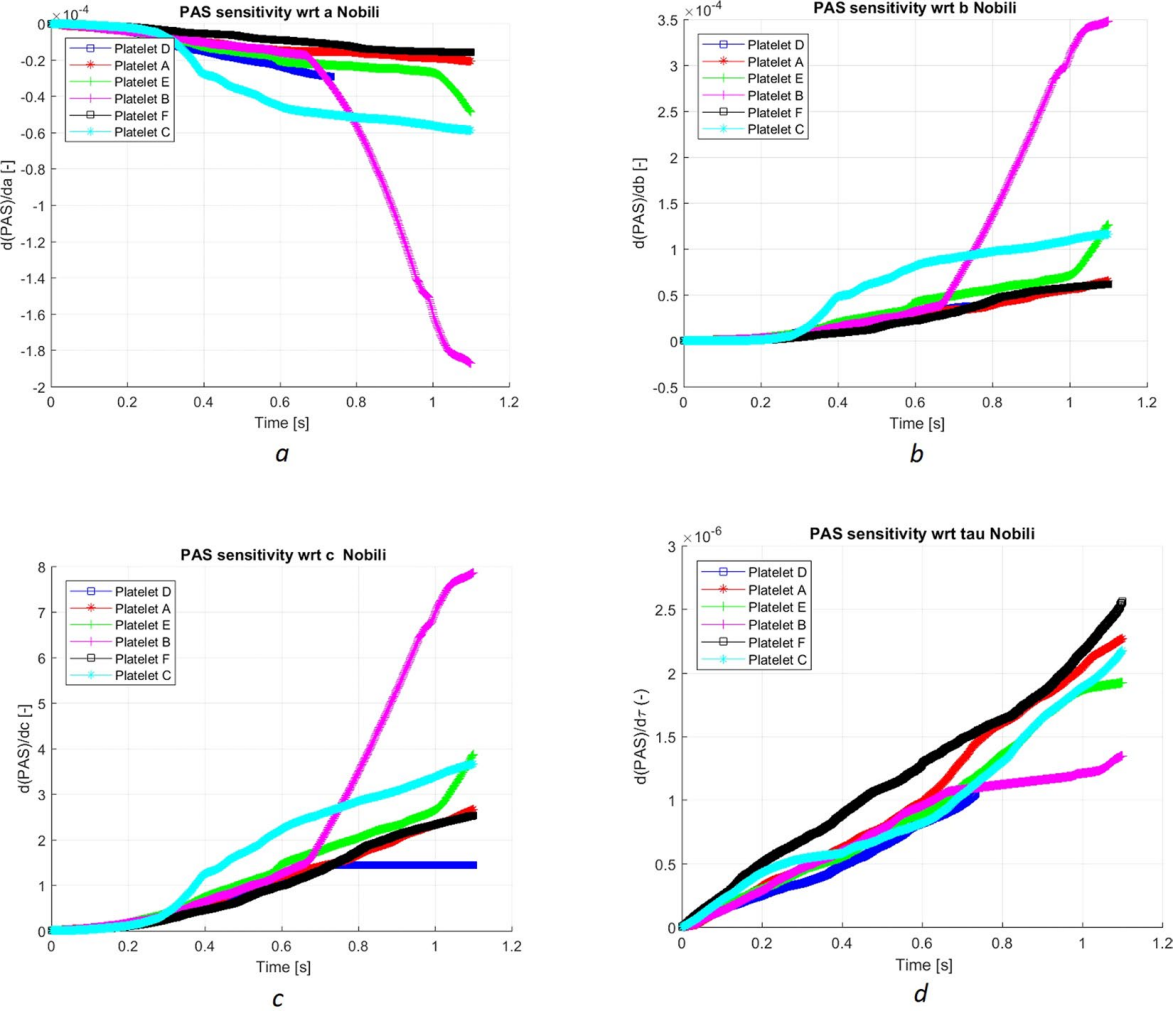
PAS sensitivity to parameters of the Nobili model. The sensitivity of PAS with respect to *a* (**a**) was smallest among the three model parameters. The sensitivity to *b* (**b**) was greater by a factor of about 2. The sensitivity to *c* (frame **c**) was more than four orders of magnitudes larger. The sensitivity to *c* is of order one, whereas the sensitivity to *a, b* and the scalar stress are of order of 10^−5^, 10^−4^ and 10^−6^, respectively. The sensitivity to the stress (**d**) is smallest. The absolute values of the derivatives are increasing in time (as for PAS) while the derivative with respect to *a* is negative. One may note that the parameters in Fig. 4a–c for Platelet B change character. Animations Video 3 and Video 7 are related to the path and time evolution of the stress experienced by Platelet B. At about 0.7 s the stress increases abruptly by almost two orders of magnitudes, occurring when the platelet enters Region II (Fig. 3).

Altogether, 10^5^ platelets were released at the inlet of the pump (about 2 pipe diameters upstream to the entrance to the pump house). According to the platelet activation models, most platelets are predicted to maintain a low level of PAS although displaying a wide range of values in the entire platelet population^11^. However, only the relatively small number of platelets characterized by a large risk for activation is of interest. Therefore, in this work, a smaller group of about 100 platelets was considered, representing the platelets subjected to large stress and/or prolonged residence times. For these platelets, each platelet path was studied, and the development of PAS was simultaneously computed by using mainly Nobili’s and Soares’ models along with the platelet pump residence time. The two models yield substantial differences in PAS for the same platelet ranging from a factor two to one order of magnitude. The PAS response of the platelet depends on its path through the pump as well as on the activation model applied. The underlying reason for the differences in PAS was due to the model parameters providing different weights to stress accumulation, local stress and residence time. The sensitivity of PAS to the model parameters in Nobili’s model (equation (11)) was considered and is presented in Fig. 4. The results found to be similar in character as for the uniform stress case.

**Figure 5:**
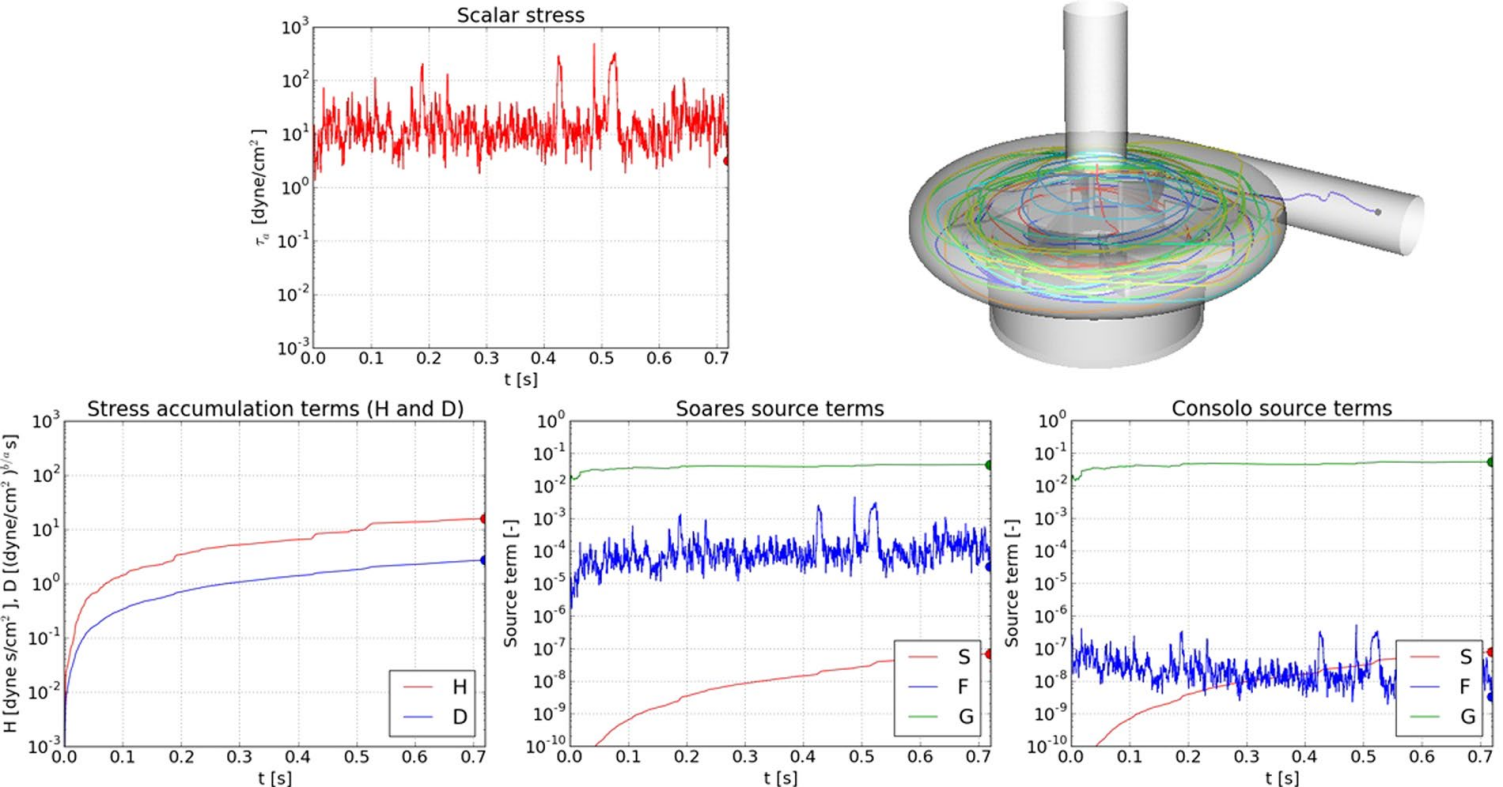
The stress and the contribution of different terms on the right hand side of the model equations (4–10) over time for Platelet A. The platelet enters in the central part of the inlet pipe (colored red) and it leaves the pump at the outlet (colored blue). The color along the platelet path is related to platelet residence time. The platelet remains in Region I (defined in Fig. 3) over a longer period of time, corresponding to about 35 pump rotations. The history term in the Nobili model (D-term) is lower than the corresponding term in the Soares and Consolo models (H-term) by construction. The Nobili model, in contrast to the Soares and Consolo models does not take into account the time-derivative of the stress. This G-term dominates over the others (S- and F-terms) by several orders magnitudes. The corresponding figures for Platelets B, C and E are given in the Supplementary material along with the animations related to the figures. Note the logarithmic scale of the variables.

The contribution of the different terms on the right side of the models (i.e. in equations (4) and (10)) was considered. In contrast to the uniform stress case, the stress in the centrifugal pump varies largely in space and time. In equations (4) and (10) the right side reflect these differences. The relative size of each term may provide insight into the model behavior and the weight that the model has in regards to the local instantaneous stress as compared to stress history. Figure 5 depict the normalized contributions of the D-, F- and S-terms of the respective models versus the evolution of PAS. In order to gain a deeper insight into the details of the behavior of the different terms, animations of the platelets motion in the pump is shown in Video 1 toVideo 4 (Supplementary material). Each animation also contains the scalar stress (τ_a_) as function of time, along the path of the platelets. Additionally, the time evolution of the different terms in the “history” (D- and H-) terms in the Nobili and Soares models, respectively, are shown. The animations show also the corresponding time-evolution of S-, F-and G- terms of the Soares and Consolo model variants, respectively. For the four platelets (A,B,C and E) the strong unsteadiness of the stress is apparent and it varies in the range of about 10 and 1000 dynes/cm^2^. By tracing the platelets and the stress signature it is possible to gain a deeper understanding of the flow. Due to the strong unsteadiness of the stress, the G-term make the major contribution to PAS, both in Soares and Comsolo models. The H-term uses the full value of the stress, whereas the D-term uses a smaller portion of it (about τ^0.47^) implying that the D-term is about one order of magnitude smaller than the H-term. The effect on the PAS development is observed in Video 5 to Video 8 (Supplementary material). The PAS predicted by the Nobili model is smaller by two to three orders of magnitudes as compared to the PAS predicted by the Soares and Consolo models.

**Figure 6:**
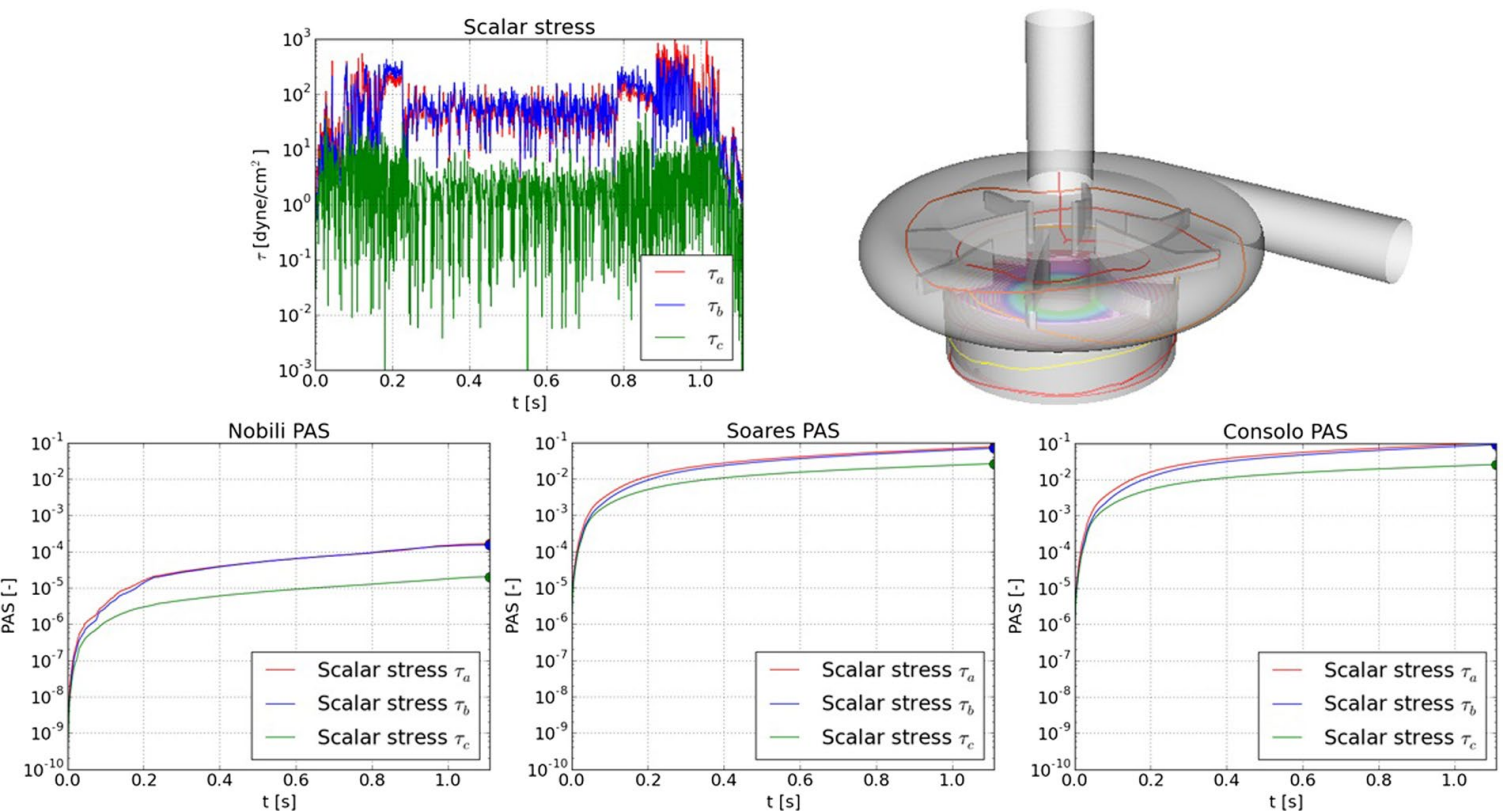
PAS as function of time (Platelet E) with the Nobili- and Soares-models, using one of the three expressions in Equation (12), τ_a_, τ_b_ and τ_c,_ respectively. The platelet stays in Region I (Fig. 3) for less than 0.2 s, where after it enters the gap between the magnet and the pump house (the Taylor-Couette gap, Region II). The platelet stays in this region for about 0.8 s when it enters Region III. The scalar stress in Region I contains contribution from both the shear- and normal-components, due to the motion of the blades. The supplementary material includes animation of the motion of the platelet. The results show that the major contribution to PAS was the large time-derivative of the stress. Corresponding results for Platelets A, B and C can be found in the Supplementary material. Note the logarithmic scale of the stress and PAS in the figure.

The predicted PAS depends on the expression for the scalar stress used by the models (equation (12)). The models use only a scalar form of the stress and do not account for stress anisotropy. Figure 6 depicts the predicted PAS for Platelet E using the three stress expressions (τ_a_, τ_b_ and τ_c,_ respectively). It is observed that the differences in PAS for a given model using different expressions for the stress can be as much as one order of magnitude, depending on the type of flow that the platelet encounters. The contribution of the normal stress was lowest in Region II while it was equally large as the shear-component in Region III.

**Figure 7:**
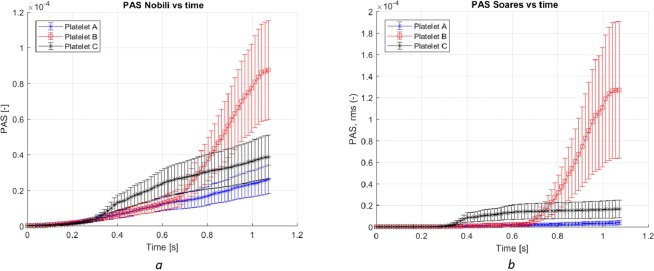
The mean and variance of PAS as function of time with the Nobili (**a**) and the Soares (**b**) model (without the G-term for the sake of comparison) applied to three typical platelets. Random parameter perturbations (10% amplitude) and 250 samples. The mean and variance of PAS was computed from the set of data for the models of Nobili and Soares. The dependence of the MC outcome was found to strongly depend on the behavior of the stress along the path of the platelet. The response of Platelet B for both models is quite similar, in contrast to the larger difference in the mean and variance of PAS for Platelets A and C.

To assess the non-linear effects of the models, the sensitivity to random perturbation in the parameters and the stress was studied using the MC approach. For each time instant, a set of the populations was computed using the randomly perturbed model parameters, calculating the mean and variance of the populations of PAS for different platelets along with their variation in time, depicted in Fig. 7. Since only the platelets at risk for activation are of interest both concerning PAS and the sensitivity, Fig. 7 depict the model response to random perturbations of 10% in the model parameters.

**Figure 8:**
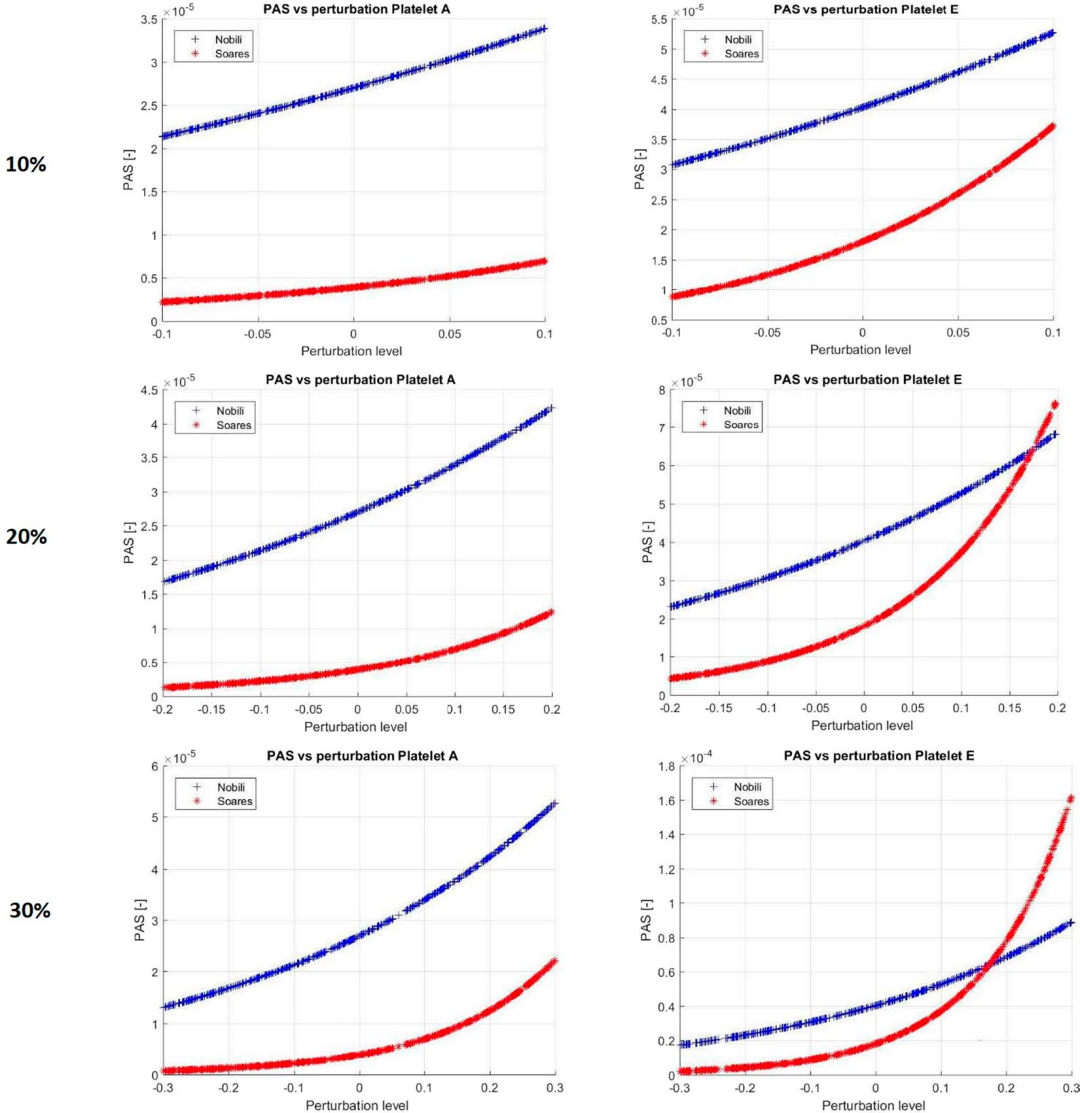
The PAS response of two platelets (right and left frames, respectively) using the Nobili (blue) and Soares’ (red) models, with perturbations with 10% (**a**,**b**), 20% (**c**,**d**) and 30% (**e**,**f**) amplitudes. The figures show that the range of PAS increases with increasing perturbation range.. The Soares’ model is more sensitive, in the sense of larger PAS deviation from the un-perturbed PAS values. The sensitivity is larger with a given positive perturbation as compared to a negative perturbation of a corresponding size. When comparing the two models, the final PAS value may be larger by a factor of 2–3 and 3–4 using Nobili’s model, using 10% and 30% perturbations, respectively. The corresponding figures for the Soares’ model indicate increase in PAS by a factor of 3–6 and by 1–2 orders of magnitudes with 10% and 30% perturbations, respectively.

The sensitivity of the models of Nobili and Soares to the level of perturbation is displayed in Fig. 8. The figure shows the variation in PAS using Nobili’s and Soares’ models by a sequence of random perturbations, having amplitudes of 10%, 20% and 30%, introduced in every time-step. Two typical platelets (denoted as Platelet A and Platelet E) are presented. Platelet A represents most platelet that leave the pump after a short time, whereas Platelet E represents platelets that stays over longer period of time in the strong shear regions. The data in the figure is related to the PAS value at the end of the simulations, (*t* = 1.1 s). The relation between PAS and the level of perturbation is generally non-linear. The relationship depends on the stress experienced by the platelet and the chosen model. The results of this MC study are in line with the semi-quantitative results obtained by analyzing the different terms of the model. The approach used here provides a quantitative, non-linear measure for the uncertainty due to the uncertainties/errors in the model parameters and in the scalar stress.

## Discussion

The sensitivity of two platelet activation models with respect to model parameters and the stress acting on the platelets were studied for a uniform stress field and the flow in a centrifugal pump. The linear sensitivity of the models was determined by computing the derivative of the PAS parameter with respect to model parameters and the scalar stress and by analyzing the contribution of the different terms in the model to the time rate of change of PAS. The results show that the sensitivity may be significant, depending on the platelet path and history. MC study of the PAS response to random perturbations of model parameters and variables were found to be rather misleading when applied to the entire platelet population. When the MC method was applied to platelets with greater activation risk, the sensitivity of the models was found to be high and increased non-linearly as the uncertainty of the parameters increased.

Platelet activation depends on the local conditions along the path of the platelet, and consequently (initially) limited to a small fraction of the platelet population. PAS expressing the portion of activated platelets in the entire platelet population may indicate a small numerical value, yet implying a higher risk for onset of thrombus formation. Platelet activation modeling has traditionally been based on arguments used for modeling of hemolysis^24,25^. However, there are major differences in the processes of hemolysis and platelet activation. Activated platelets, in contrast to damaged red blood cells, may lead to activation of more platelets through a strong biochemical amplification and feedback mechanism. Here, we interpret PAS as a measure related to the probability of activating a specific platelet. This interpretation may not be in complete agreement with the experimental data used to calibrate the activation models, where PAS is related to the entire platelet population. Yet, considering the path of groups of platelets at greater risk of being activated, provides an idea of how the details of the flow (stresses and residence times) affect the platelets in different areas within the centrifugal pump. Thereby the understanding of the behavior and potential shortcomings of the activation models can be improved.

The linear sensitivity analysis of the models gives insight into the models, and in particular at the initial stages (low PAS values) of activation. For example, the PAS derivative with respect to parameter *a, i.e*. $d(\partial \,PAS/\partial \,a)/dt$ in equation (11a), turns out to initially be negative for most cases, due to the first term in the bracket on the right hand side ($-(b\,\mathrm{ln}\,\,\tau )/{a}^{2}$). This term remains negative, unless the stress is less than about 16 dyn/cm^2^. This behavior may be reasonable for slow and stagnant flows. The contribution of the different terms to the time-derivative of PAS during the platelet journey is instructive to achieve a better understanding of the models. The D-term in equation (4) is large and is “balanced” by the small size of the *c*-parameter (10^−5^). The size of the explicit stress (in form of ${\tau }^{b/a}$) is also several orders of magnitude smaller than the D-term. The need for using a very small value for *c* may be related to the observation made about the inconsistency of the model (when *b/a* differs from one)^12,24^ where D is related to stress accumulation in a nonlinear manner. Reducing the parameter *a* to a value below unity would imply change of signs in the last term in equation (11a) and (b).

In the Soares’ model (equation (10)), the F-term dominates over the S-term. The S-term accounts for the history effect of the stress on the platelet, similar to Nobili’s D-term. The strong unsteadiness of the flow in the pump implies that the G-term (averaged time-derivative of the stress) of both the Soares’ model and Consolo’s version, dominates over the other terms (Fig. 5 and the Supplementary material). The F- and G-terms would be of the same order only when the stress is slowly varying, since the parameters α,β and γ,δ are of roughly the same size. The G-term is always positive and hence it contributes additively to PAS. The accumulative effect contribution of the stress fluctuations, which is known to affect the platelet and its “sensitization”, was left out from the models. Future models should take into account also the accumulated contribution of the temporal variations of the stress.

Soares’ model uses the SA and the SR parameters (equation (13)). The former, SA, enters in the S-term whereas SR enters into the G-term (equation (9)). These two parameters behave in a similar manner, except at the initial stages when the smoothing effect of accumulation is less prominent. It was noted that the SR-parameter was larger than SA by a factor of about four orders of magnitudes, indicating the strong time variations of the flow in the centrifugal pump. Moreover, high SR values imply large increase in τ, promoting a more pronounced increase in PAS.

The numerical values of the individual model parameters may play a significant role for model behavior. During the analysis a difficulty was observed regarding the Soares’ model due to the inclusion of the stress-rate term (G, in equation (9)). The initial behavior of PAS near *t* = 0 (with PAS = 0), depends on the β and δ parameters. A significant effect of the initial condition can be observed when changing the value of β from 1.4401 (Soares) to 0.72005 (Consolo). At the initial stages near *t* = 0 (PAS vanishes) the contribution from the S-term (equation (7)) to the time development of PAS is small since H (equation (6) is also small. The contribution from the F-term is proportional to $PAS{(t)}^{\beta -1/\beta }$, implying that the initial behavior of PAS depends on (β−1)/β. For the original Soares’ model, the exponent is positive ( = 0.7457) while the corresponding value for the Consolo’s model the exponent is negative ( = −0.6687). Thus, the dependency of time derivative of PAS initially is large for the Consolo’s model whereas it is smooth for the original Soares’ and Nobili’s models. This problem can be overcome by requiring that β > 1 or starting with a non-vanishing PAS. Similar reasoning may be applied to the G-term (equation (9)). The numerical value in both Soares’ and Consolo’s models was δ = 0.5125, whereas different γ values were suggested, as noted in Table 1. The initial behavior of equation (10), due to the G-term, would be directly proportional to $PAS{(t)}^{\delta -1/\delta }$. Thus, the initial growth of PAS could be very large when *δ* < *1*. This implies an initial very strong temporal PAS growth in fields whenever SR is large (as indicated by the initial PAS slope in Fig. 1c. This behavior could be remedied by choosing a larger initial value for PAS and preferably using a model parameter such that *δ* > *1*. The longer term effect of the initial PAS value is less pronounced as the G-term is large in the pump and thereby dominates the temporal evolution of PAS.

Applied to the pump case, the MC approach show that the variance of PAS over the entire populations exhibit lower spreading as compared to the uniform stress field case. For example, when a pump running at 2,500 rpm and 3.1 L/min were subjected to a 5% random perturbation the mean and variance of PAS over the whole populations were 1.3∙10^−4^ and 9.4∙10^−4^, respectively. On the other hand, for some individual platelets the corresponding extreme PAS values are not reflected by the variance. Yet, from the point of view of risk for thrombosis, the activation risk of individual platelets should be considered most important. Therefore, to assess the risk for platelet activation, the median and extreme values of PAS should be used rather than the corresponding mean and variance of PAS.

The current results also show a significant dependence on the expression used for the scalar stress. The models considered here do not account for largely anisotropic stress. Recent studies^7,8^ indicate that platelets respond to non-isotropic stress (mechano-sensing). As it is experimentally very difficult to measure the components of the stress tensor in flowing blood, detailed flow simulations could potentially be used for improved model development. From such simulation it may be possible to derive expressions for the effects of the stress components on platelets as observed in macro-scale. The effects of the forces acting on the platelets, in form of reversible and irreversible changes^26^ should also be accounted for in future activation model.

The PAS models considered here are calibrated for a relatively low stress cases. A recently published work^11^ showed that high shear-rates may be found in centrifugal pumps. In such regions thrombi may be formed not by direct platelet activation but rather by the unfolding of the vWF which aggregates platelets without initial platelet activation^22^. This aspect should be accounted for in future thrombus formation modeling.

The activation models considered here, were formulated in a dimensional form. The left hand side of the models has a dimension of s^−1^ whereas the dimensions of the terms on the right hand side are adjusted by model parameters. By recasting the models into a dimensionless form, the generality and robustness of the model can be enhanced regarding parameter errors. The dimensional quantities used in the models are the stress and time. These quantities may be normalized in different ways reflecting the importance of some specific processes. For example, time may be normalized by “wall” time, flow related time (D/U, with D and U being length scale and velocity in the pump, respectively), shear-time (i.e. shear rate defined as stress divided by the fluid viscosity), a “platelet-time” based on the response time of the platelet skeleton or the reaction time of the platelet membrane to stress. Similarly, the strain-rate can be used instead of the stress, assuming constant viscosity, without the need to introduce a stress scaling. It is also possible to use a stress scaling based not on the fluid viscosity but rather the modulus of the platelet skeleton and membrane. In general, recasting the dimensional quantities used in equations (4)–(10) into dimensionless forms and thereby reflecting a better insight into the importance of different quantities used by the model. Another advantage of using dimensionless quantities is the potentially larger range of model validity and avoiding model inconsistency of the power law model as pointed out by Soares *et al*.^14^.

Using a stress accumulation effect in form of a monotone increasing function may be non-physical (D and H in equations (4) and (6), respectively). Accounting for the stress effects on membrane permeability of the platelet (possible partial activation) should also be considered, but such effects are transient and at least partially reversible^10^. Lastly, the accumulated stress should consider the stress in a “time-weight” form, such that the effect of “old” stress would diminish with time. The time-scale for the stress accumulation should then be related to the restitution time of the membrane permeability.

In spite of the shortcomings found, the models discussed here do give a rough qualitative indication to the locations corresponding to sites where thrombi are observed in clinic^11^. The sensitivity of the models indicate the extent of acceptable deviation from model calibrating conditions. Hence, despite the strong model sensitivity, the phenomenological models investigated are important to obtain an indicative and qualitative understanding of the thromboembolic behavior of blood carrying devices. The results presented and the conclusions drawn for PAS based models are applicable also to other power law models, and in particular to frequently used models for predicting hemolysis. The strong sensitivity of models of hemolysis was discussed by Yu *et al*.^24^ The paper of Yu *et al*. showed that Hemolysis Index was found to vary by several orders of magnitudes among the different models (and experimental data) investigated. Model improvements could make the models to become a quantitative tool that would help in designing future devices.

## Supplementary information

Supplementary material

Supplementary Video 1

Supplementary Video 2

Supplementary Video 3

Supplementary Video 4

Supplementary Video 5

Supplementary Video 6

Supplementary Video 7

Supplementary Video 8
