# Supplementary material for: Modeling sensitivity and uncertainties in platelet activation models applied on centrifugal pumps for extracorporeal life support
Source: Sci Rep. 2019 Jun 19;9:8809. doi: 10.1038/s41598-019-45121-2 (PMC6584555; doi:10.1038/s41598-019-45121-2)
Supplement: Supplementary file 1 — Supplementary material [file 41598_2019_45121_MOESM1_ESM.pdf]

# Modeling sensitivity and uncertainties in platelet activation models applied on centrifugal pumps for extracorporeal life support

Gabriel Fuchs, Niclas Berg, L. Mikael Broman & Lisa Prahl Wittberg

## Supplementary material

The supplementary material contains animations of the path of some platelets in the Centrimag blood pump along with the time-evolution PAS, the terms in three models as function of the expression used for the scalar stress. The different regions of the pump are depicted in Fig. 3. The details of the simulations and the properties of the flow field are given in Ref 13. Operating conditions of the pump: 4 L/min at 3,000 rpm.

**Video1**

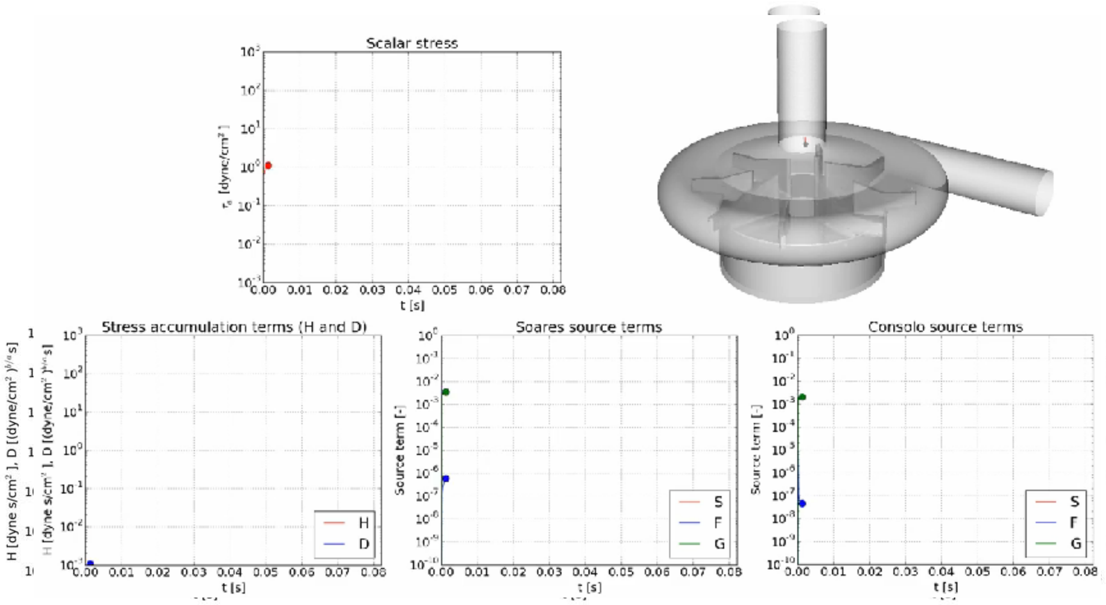


**Figure S1**

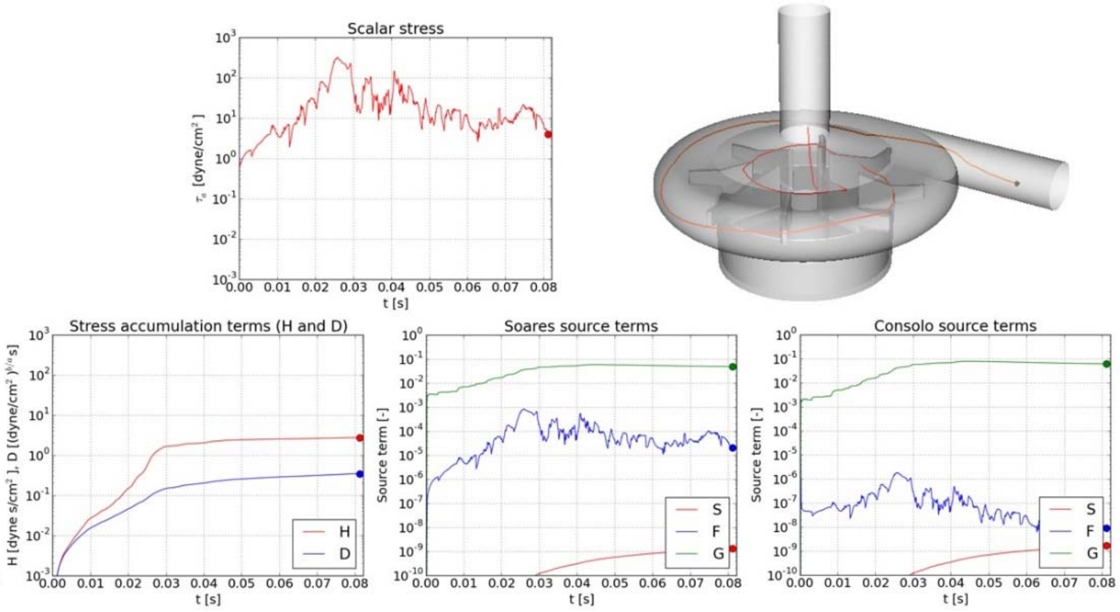


The motion of *Platelet C* in the pump. The platelet stays altogether 0.08s in the pump. It makes about 2 turns while staying on Region I. The level of the scalar stress increases as the platelet moves from the entrance into the blade region. The H-term is always larger than the corresponding D-term due to the exponent of *b/a* (=0.47) in equation (4). The G-term is large since the Stress Rate (equation (13)) contains the time-derivative of the stress. The platelets are exposed not only to turbulence (which is mostly smoothed out by the definition of SR) but also to strong larger scale structures generated by the blades. Due to the short residence time of the platelet the S-term contribution is small. Note the logarithmic scale that is used for all variables in the video.

**Video 2**

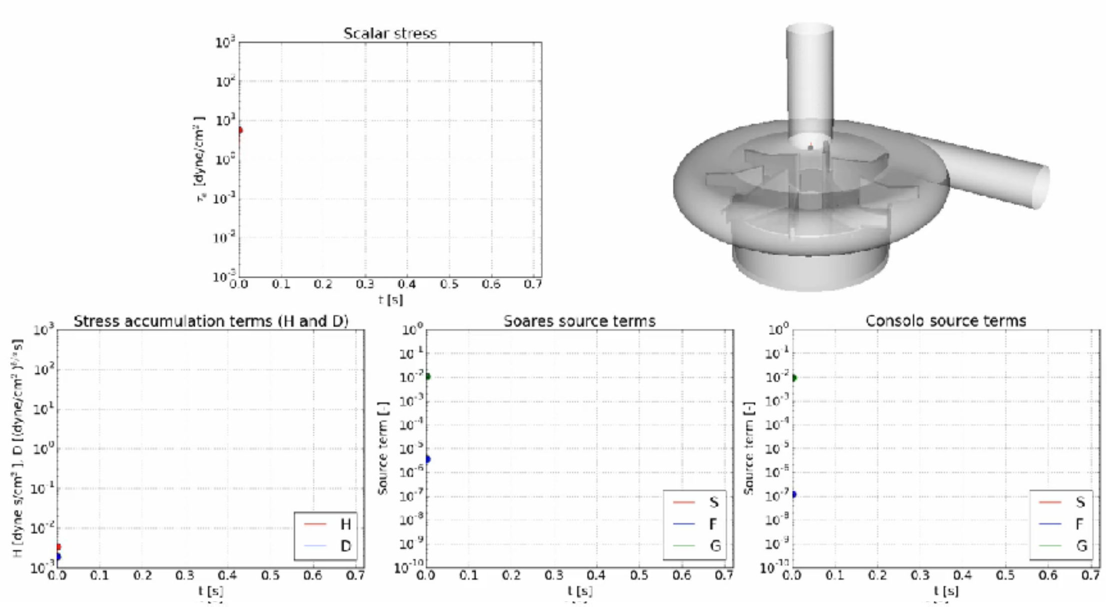


**Figure S2**

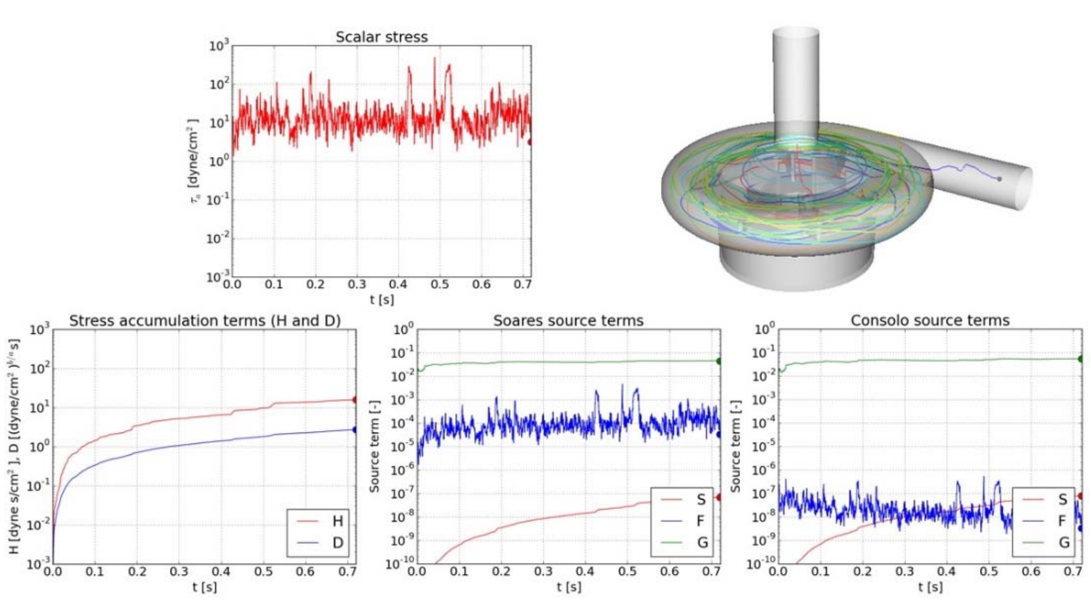


The motion of *Platelet A* in the pump. This platelet represents most of the platelets of the population as the members of this group stay relatively short time in the pump (0.7s) and they stay in the region between the rotating blades and the roof of the pump. The residence time of this platelet is equivalent to about 35 pump rotations. During this period the *G*-term in both Soares and Consolo models are roughly the same and are much larger than the components.

**Video 3**

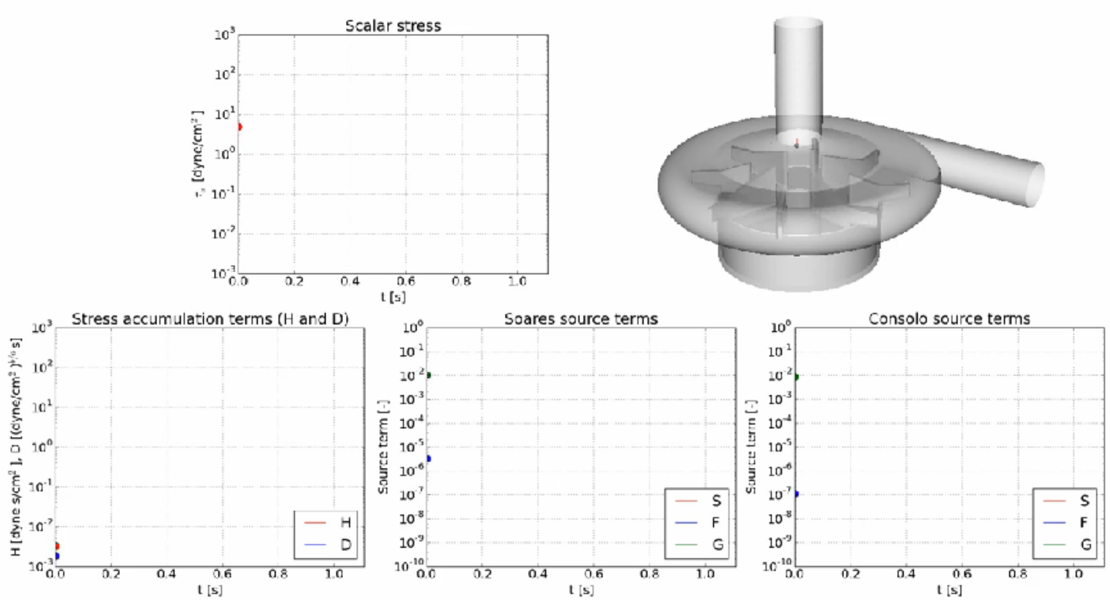


**Figure S3**

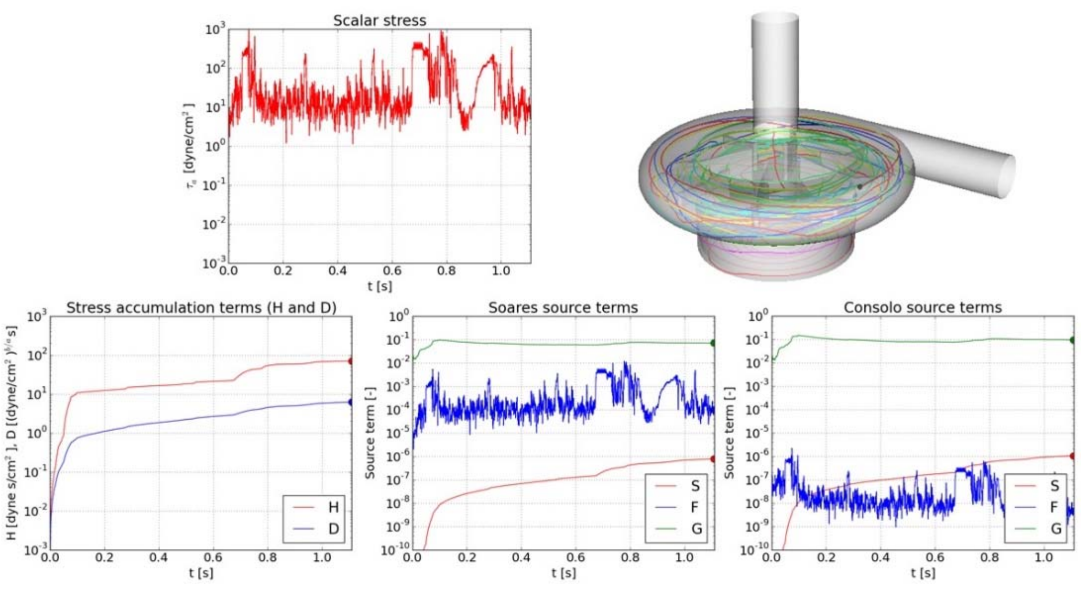


The motion of *Platelet B* in the pump. This platelet remains in the Region I (Fig. 3a) until about 0.65s when it enters Region II (Taylor-Couette region) where it encounters very strong shear-stress (increase by almost two orders of magnitude). The platelet stays in the pump throughout the simulation (1.1s) which is equivalent to about 55 pump rotations. The F-term in the Soares model is much larger than in the Consolo variant due to the value of *b*. The Consolo model has a negative exponent on PAS (equation (8)) with, b=0.72, implying that (b-1)/ b =-0.69. The corresponding exponent in Soares model (b=1.44) is (b-1)/ b =0.75. The *G*-terms dominate in both models, though it is somewhat larger in the Soares model.

**Video 4**

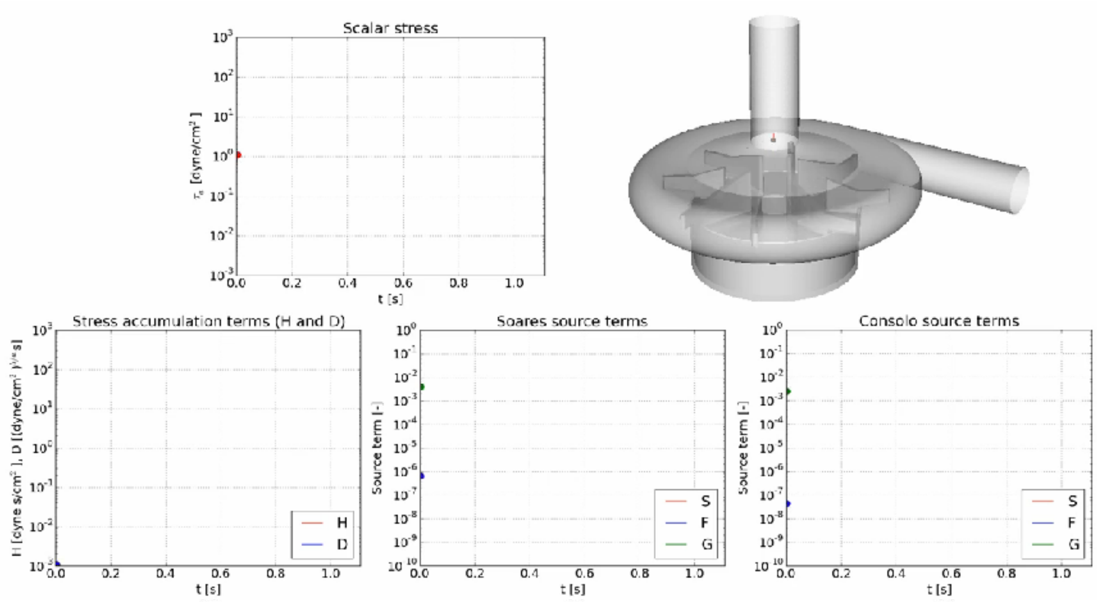


**Figure S4**

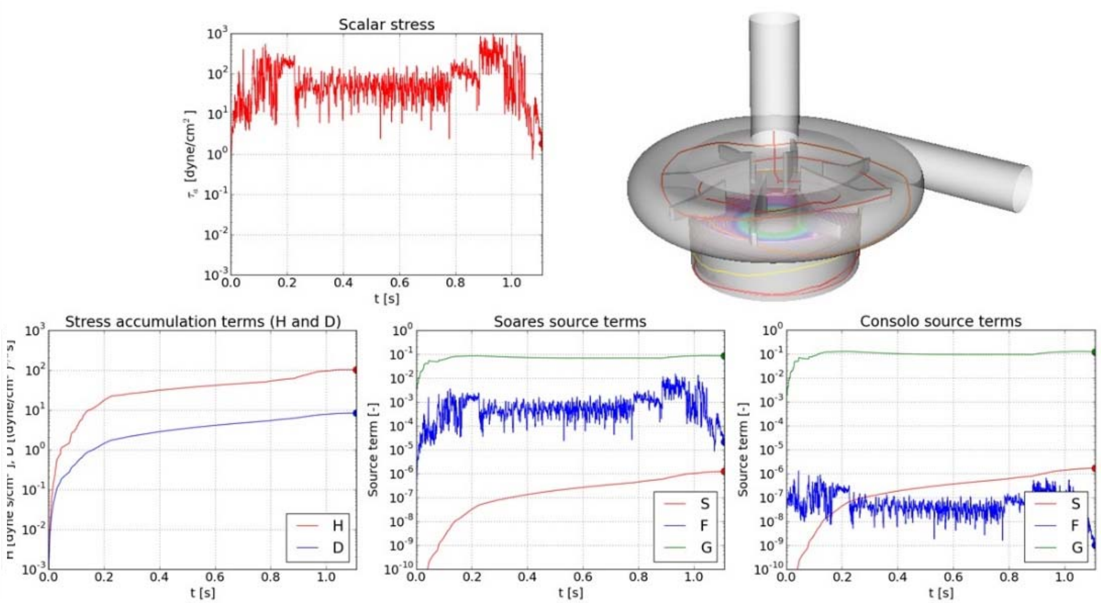


The motion of *Platelet E* in the pump. This platelet remains in the Region I (Fig. 3a) during a very short time (< 0.2s) when it enters Region II (Taylor-Couette region) where it encounters strong shear-stress. The normal-stress in this region is smaller than the shear-stress by almost two orders of magnitudes. The platelet enters Region III at about 0.95s leading a decrease in the stress towards the end of the simulation. The contribution of the different terms to PAS is mainly due to the G-terms. In this case the Consolo model yields a somewhat larger G as compared to the Soares model. The F-term contributes more in the Soars model as is explained in the legend of Video3.

**Video 5**

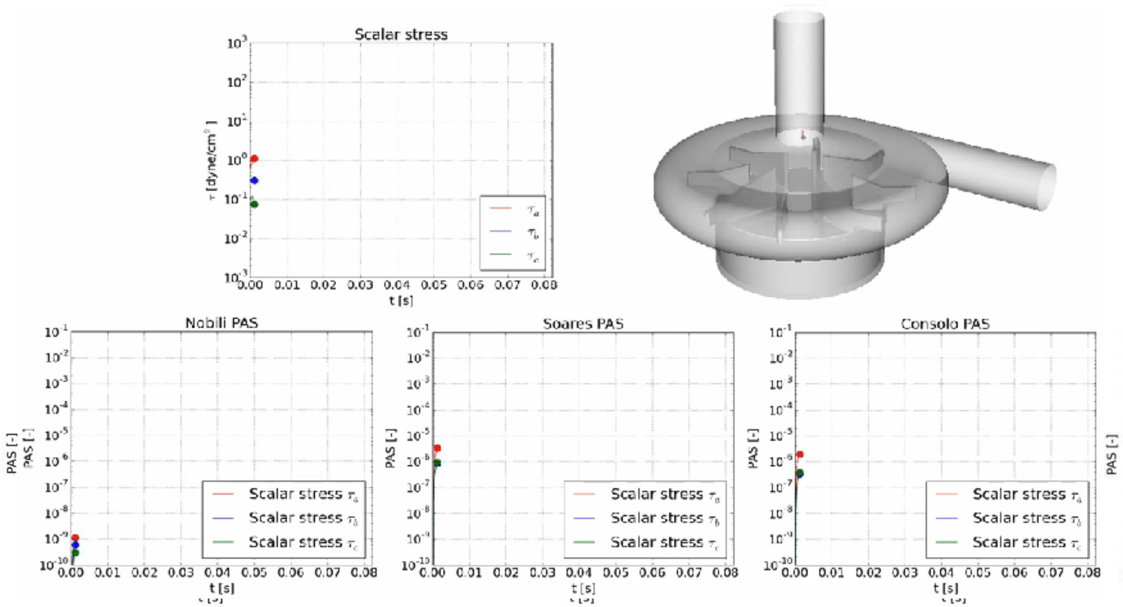


**Figure S5**

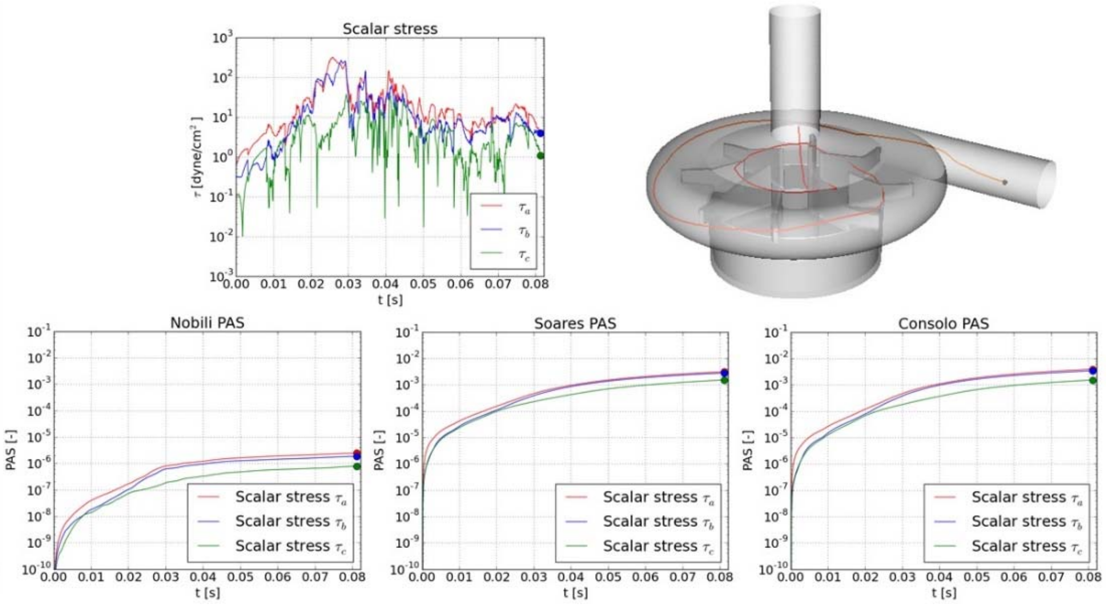


The motion of *Platelet C* in the pump. The platelet stays altogether 0.08s in the pump. It makes about 2 turns while staying on Region I. The level of the scalar stress increases the platelet moves from the entrance into the blade region. The normal stress is smaller than the shear stress in some regions but it can be as large as the shear-stress in others. Generally, the shear components of the stress is larger than the normal components, except at the initial stage (<0.02s). The PAS value due to the Nobili model is considerably lower than those predicted by the Soares and Comsolo models. In all cases, when the scalar stress uses only the normal components, the PAS value is lower than the value obtained when using the shear-stress or all the components of the stress tensor. The PAS value computed by the different models is lower as compared to the other platelets in the supplementary due to the short residence time. Note the logarithmic scale that is used for all variables in the video.

**Video 6**

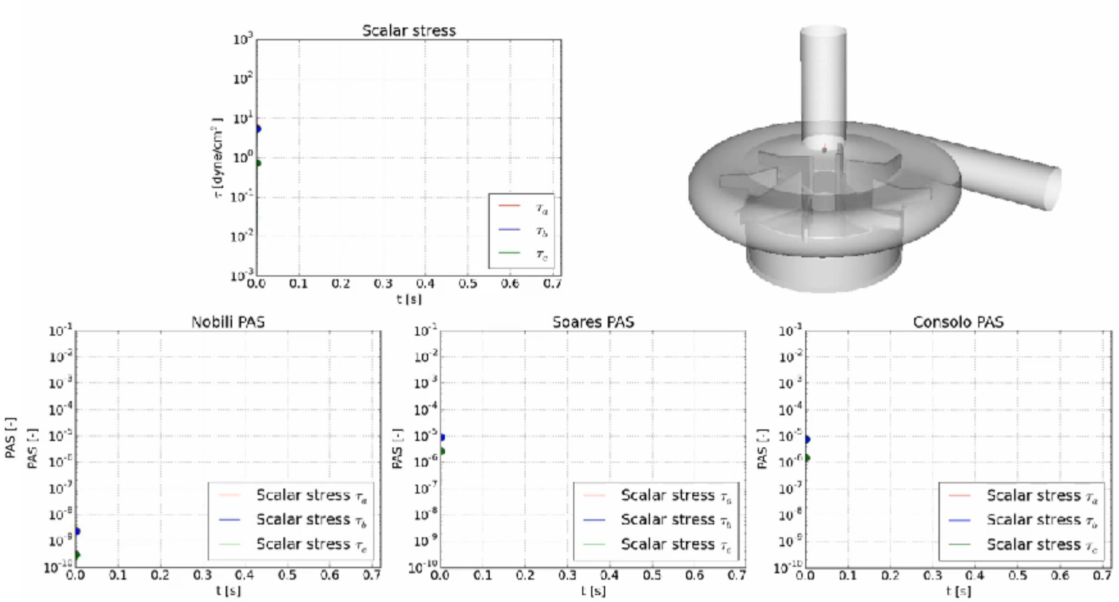


**Figure S6**

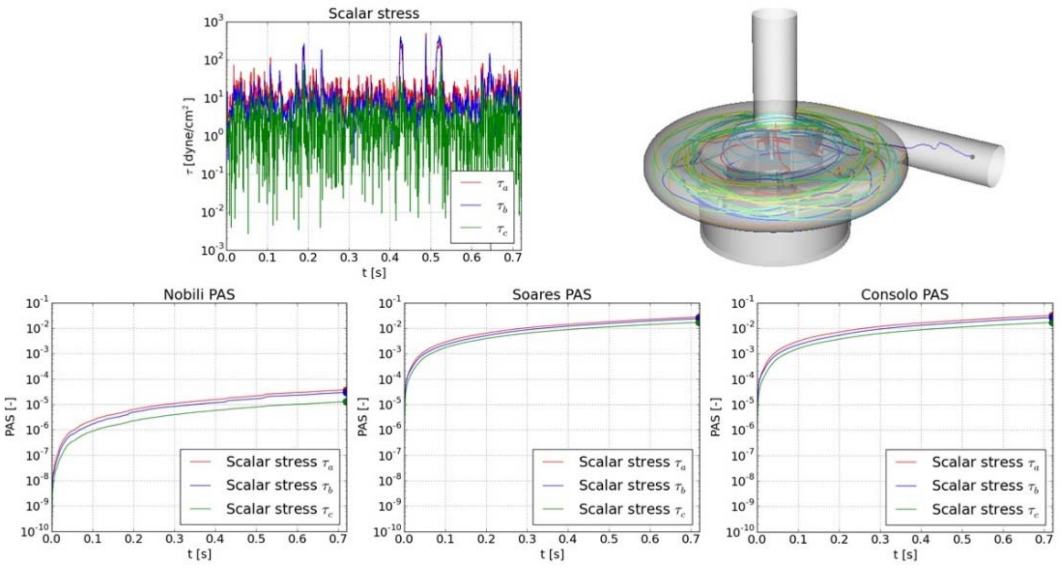


The motion of *Platelet A* in the pump. This platelet represents most of the platelets of the population as the members of this group stay relatively short time in the pump (0.7s) and they stay in the region between the rotating blades and the roof of the pump. The expression of the scalar stress differs instantaneously but the total effect on PAS, within each model is relatively small. The difference between in PAS the Soars and Consolo models are small.

**Video 7**

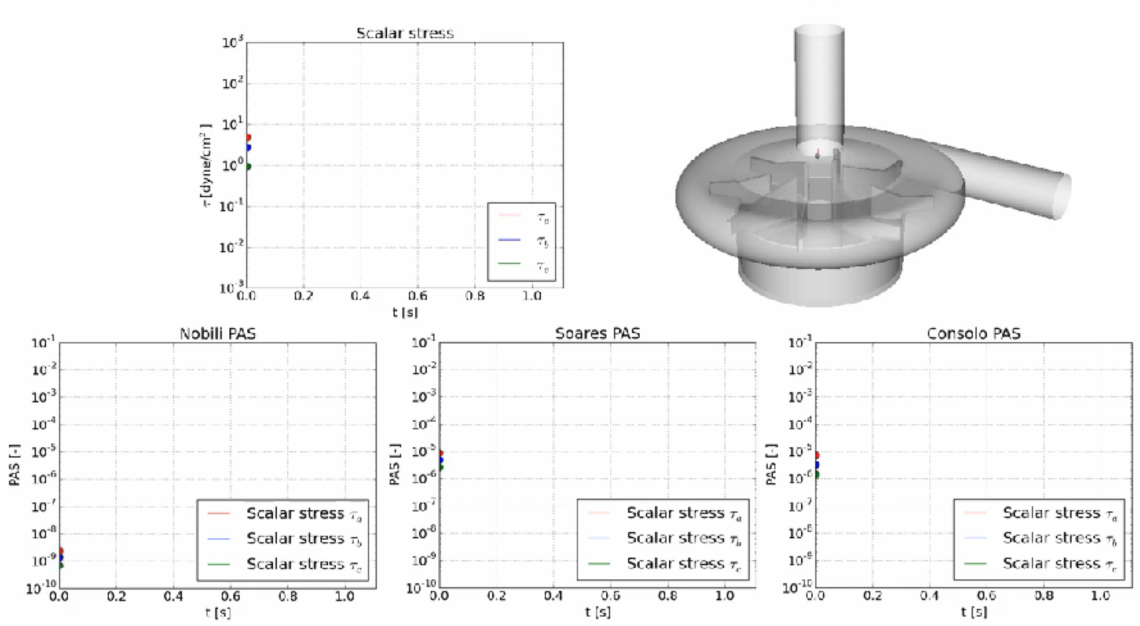


**Figure S7**

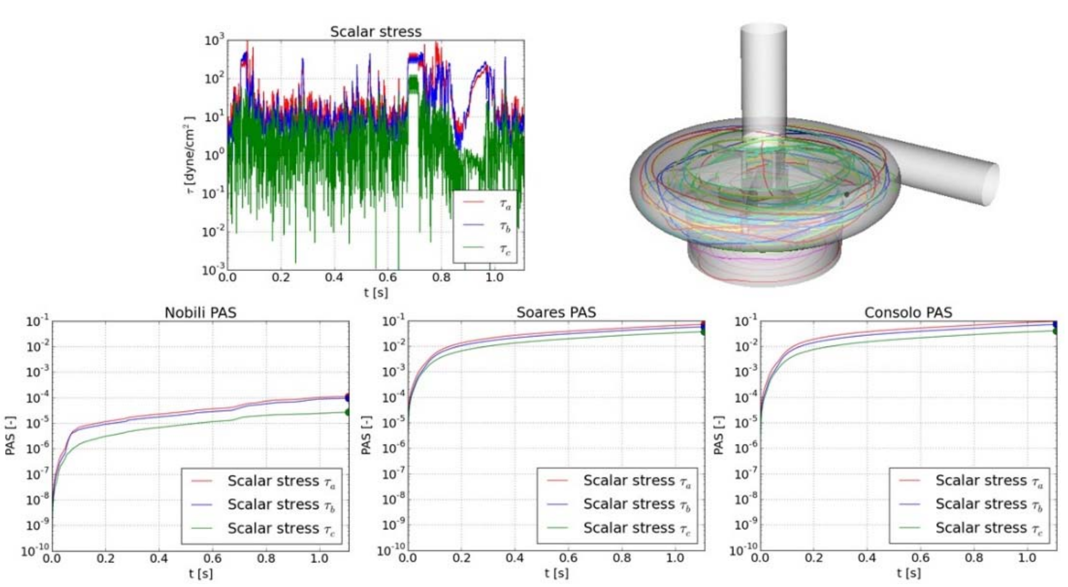


The motion of *Platelet B* in the pump. This platelet remains in the Region I (Fig. 3a) until about 0.65s when it enters Region II (Taylor-Couette region) where it encounters very strong shear-stress (increase by almost two orders of magnitude). The platelet stays in the pump throughout the simulation (1.1s) which is equivalent to about 55 pump rotations. During about 0.9s – 1s there is a significant (factor of more than 100) decrease in the normal stress components. During this period the platelet is circulating near the inner (rotating) wall of the magnet house. Before and after this period the platelet is closer to the outer wall where the centrifugal force generates a stronger normal stress. The Consolo model yields lower PAS with the normal stress components (i.e. higher sensitivity). The same model predicts larger PAS as compared to the other model.

**Video 8**

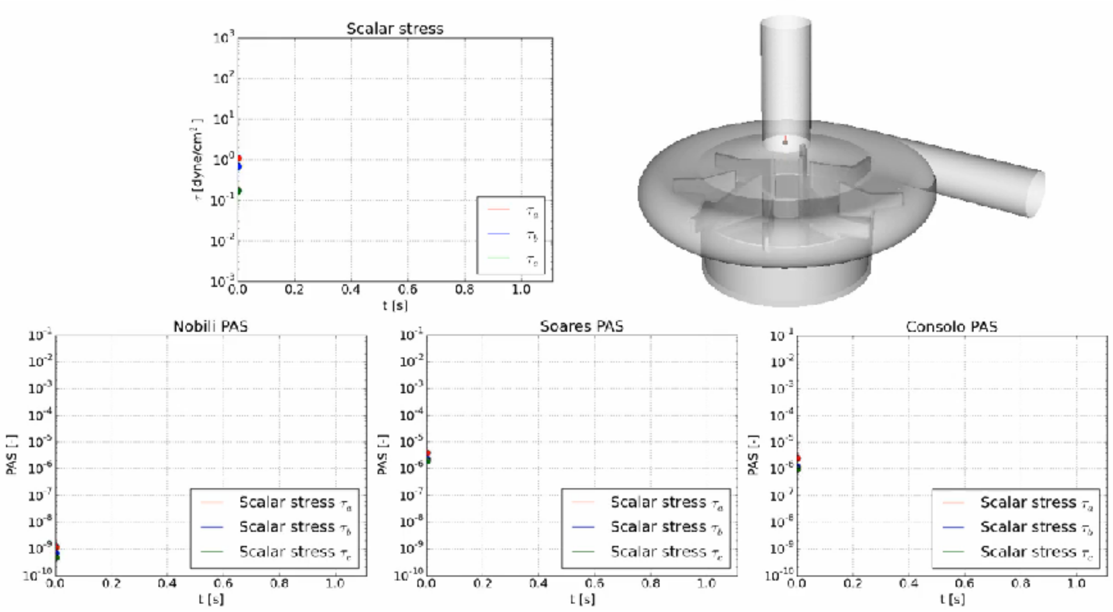


**Figure S8**

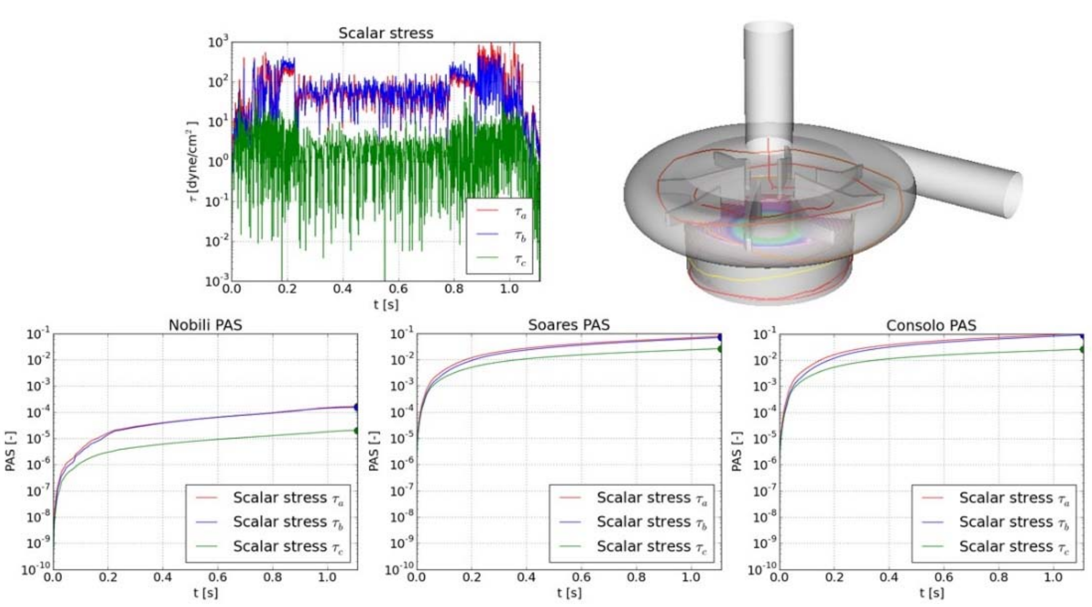


The motion of *Platelet E* in the pump. This platelet remains in the Region I (Fig. 3a) during a very short time (< 0.2s) when it enters Region II (Taylor-Couette region) where it encounter a strong shear-stress. The normal-stress in this region is smaller than the shear-stress by almost two orders of magnitudes when the platelet stays closer to the inner wall. The platelet enters Region III at about 0.95s leading a decrease in the stress. The overall effect of the platelet path and the stress that it encounters is that the Consolo model predicts largest PAS value with the common scalar stress definition. When the normal-stress components are used the PAS value is larger with Soares model.
